# The histone acetyl transferases CBP and p300 regulate stress response pathways in synovial fibroblasts at transcriptional and functional levels

**DOI:** 10.1038/s41598-023-44412-z

**Published:** 2023-10-10

**Authors:** Monika Krošel, Marcel Gabathuler, Larissa Moser, Malgorzata Maciukiewicz, Thomas Züllig, Tanja Seifritz, Matija Tomšič, Oliver Distler, Caroline Ospelt, Kerstin Klein

**Affiliations:** 1 Center of Experimental Rheumatology, Department of Rheumatology, University Hospital Zurich, University of Zurich, Zurich, Switzerland; 2 Department of Rheumatology, University Medical Centre Ljubljana, Ljubljana, Slovenia; 3 Faculty of Medicine, University of Ljubljana, Ljubljana, Slovenia; 4 Department of BioMedical Research, University of Bern, Murtenstrasse 28, 3008 Bern, Switzerland; 5 Department of Rheumatology and Immunology, Inselspital, Bern University Hospital, Bern, Switzerland

**Keywords:** Rheumatoid arthritis, Acetylation, Proteasome, Molecular biology, Histone post-translational modifications, Autophagy

## Abstract

The activation of stress response pathways in synovial fibroblasts (SF) is a hallmark of rheumatoid arthritis (RA). CBP and p300 are two highly homologous histone acetyl transferases and writers of activating histone 3 lysine 27 acetylation (H3K27ac) marks. Furthermore, they serve as co-factors for transcription factors and acetylate many non-histone proteins. Here we showed that p300 but not CBP protein expression was down regulated by TNF and 4-hydroxynonenal, two factors that mimic inflammation and oxidative stress in the synovial microenvironment. We used existing RNA-sequencing data sets as a basis for a further in-depth investigation of individual functions of CBP and p300 in regulating different stress response pathways in SF. Pathway enrichment analysis pointed to a profound role of CBP and/ or p300 in regulating stress response-related gene expression, with an enrichment of pathways associated with oxidative stress, hypoxia, autophagy and proteasome function. We silenced CBP or p300, and performed confirmatory experiments on transcriptome, protein and functional levels. We have identified some overlap of CBP and p300 target genes in the oxidative stress response pathway, however, with several genes being regulated in opposite directions. The majority of stress response genes was regulated by p300, with a specific function of p300 in regulating hypoxia response genes and genes encoding proteasome subunits. Silencing of p300 suppressed proteasome enzymatic activities. CBP and p300 regulated autophagy on transcriptome and functional levels. Whereas CBP was indispensable for autophagy synthesis, silencing of p300 affected late-stage autophagy. In line with impaired autophagy and proteasome function, poly-ubiquitinated proteins accumulated after silencing of p300.

## Introduction

The activation of stress response pathways is an early event in joints of patients with rheumatoid arthritis (RA). In particular, adaption to hypoxia and oxidative stress, autophagy, the unfolded protein response (UPR) and the endoplasmatic reticulum (ER) stress response are activated pathways in the RA synovium^1–6^.

Synovial fibroblasts (SF) are the key joint resident cells that create a joint-specific microenvironment^7^ and drive joint destruction and inflammation in RA^8^. Furthermore, SF promote angiogenesis by producing vascular endothelial growth factor (VEGF)^8^. The neovascular network is dysfunctional, rendering the synovial tissue hypoxic. Hypoxia and subsequent oxidative stress within the RA joint significantly contribute to the activation of SF and induce a metabolic switch from oxidative phosphorylation towards glycolysis^9^. This is associated with an increased invasive behavior and proliferation of SF, and the secretion of chemotactic factors that lead to migration of inflammatory cells^1,10^. The upregulation of metabolic and stress response genes was described as a characteristic of SF in chronic versus acute inflammation in a mouse model upon repeated exposure to inflammatory stimuli^11^.

Upstream regulatory factors that integrate the stress and inflammatory response in RA SF have not been investigated yet in detail. CREB-binding protein (CBP) and p300 are two highly homologues enzymes with histone acetyltransferase (HAT) activities. They are often considered as proteins with identical functions (referred as CBP/ p300) and few studies address their individual roles^12–14^. CBP/ p300 are widely studied as writers of the activating histone mark histone 3 lysine 27 acetylation (H3K27ac) but many non-histone proteins are among their targets, including several transcription factors^15,16^. We have recently studied individual functions of CBP and p300 in regulating the inflammatory response of SF^14^. We have identified p300 as the major HAT in SF that possessed overlapping and distinct target genes and functions with CBP^14^.

Here we focused on the regulation of CBP and p300 expression and their individual roles in regulating stress response pathways in SF. We used existing transcriptome analysis of unstimulated and TNF-stimulated SF silenced for CBP or p300, respectively. We performed an in-depth analysis on their contribution in regulating different stress response pathways in SF and performed confirmatory experiments on RNA, protein and functional levels.

## Results

### p300 expression is suppressed by TNF and oxidative stress

**Figure 1:**
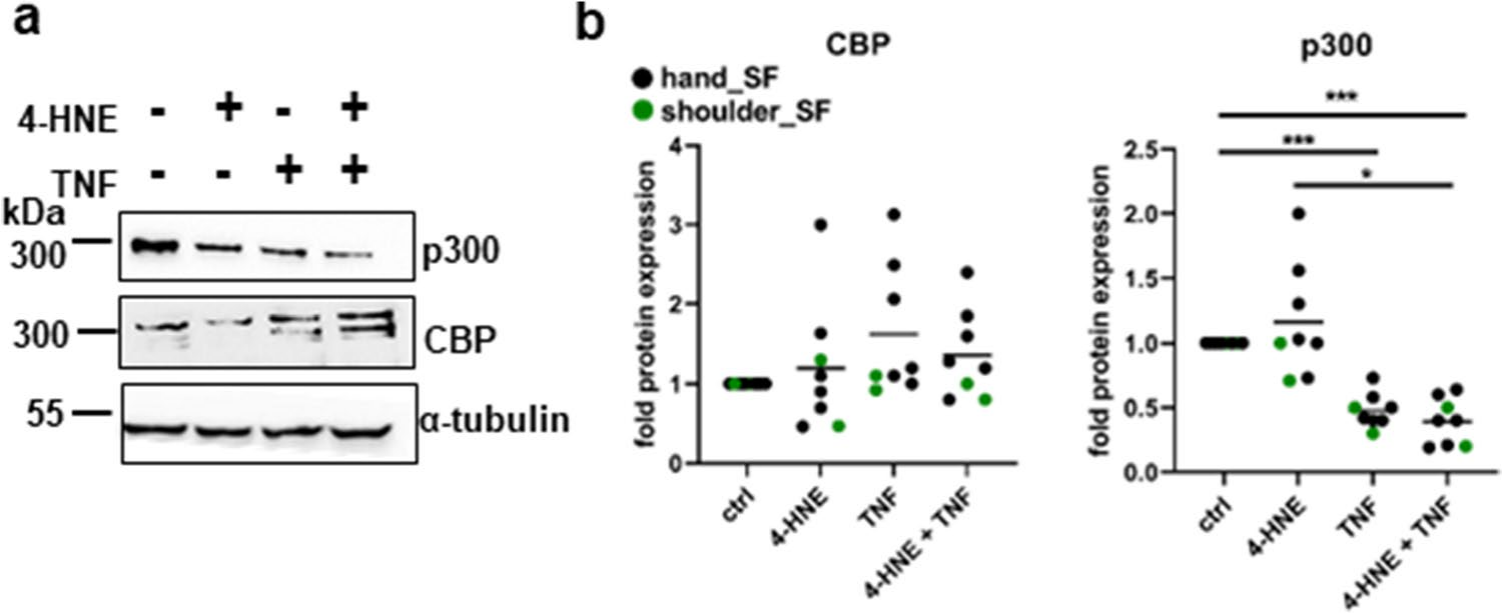
Regulation of CBP and p300 protein expression. **(a)** Representative Western blot and **(b)** densitometric analysis of SF (n = 8) treated with 4-HNE and TNF, or a combination of both. Full length blots are shown in supplementary Fig. 1 *p < 0.05, ***p < 0.005.

To study CBP and p300 regulation, we have treated SF with the lipid peroxidation products 4-HNE to mimic oxidative stress^17^, in absence and presence of TNF. Whereas 4-HNE and TNF did not induce any consistent effects on the expression of CBP, the expression of p300 was suppressed by TNF by 52,1% (± 13,2%) and was even further down regulated in presence of TNF and 4-HNE by 60,7% (± 18,0%) (Fig. 1a, b, supplementary Fig. 1). In addition, the expression of p300 but not CBP was dose-dependently suppressed by H_2_O_2_ (supplementary Fig. 2). To study individual functions of CBP and p300, we followed a silencing strategy for both enzymes, enabling us to compare the effects of p300 to those of CBP. Our results on p300 regulation suggest, that the silencing approach for studying p300 function, resembled p300 expression in SF under conditions of oxidative stress and inflammation (Fig. 3d).

### CBP and p300 regulate stress response pathways in SF

**Figure 2:**
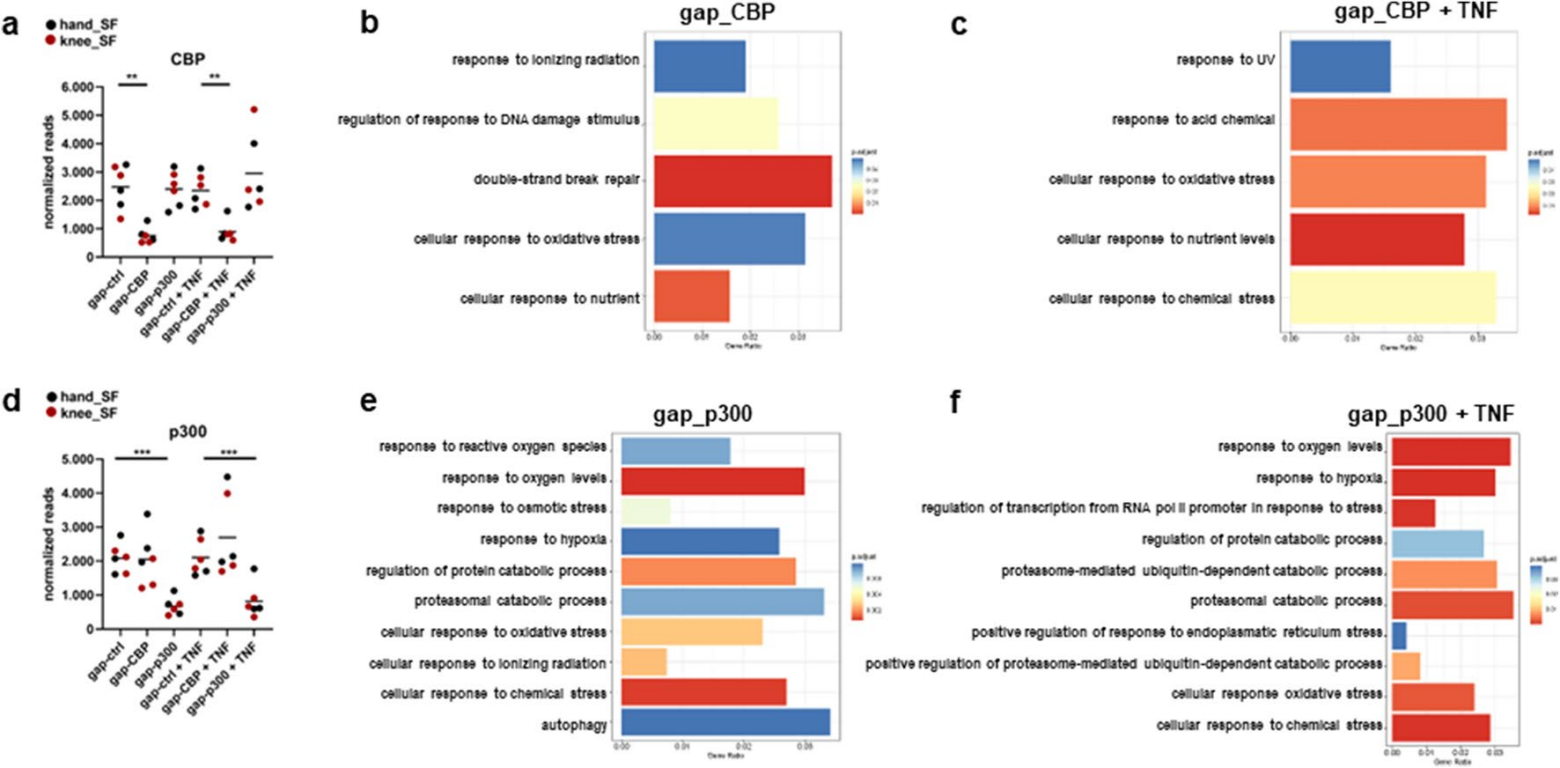
CBP and p300 target genes are enriched in stress response pathways. Transcriptomes were determined by RNAseq^14^. Normalized reads confirming the silencing of **(a)** CBP and **(d)** of p300. Significantly affected genes (± fold change > 1.5, FDR < 0.05) entered pathway enrichment analysis for Gene Ontology (GO) biological process (BP). Enriched GO BP identified after silencing of **(b)** CBP and **(e)** p300 in absence and **(c)** and **(f)** presence of TNF. Details on displayed pathways are shown in supplementary table 2. **p < 0.01, ***p < 0.005.

We used existing RNAseq data sets^14^ from knee and hand SF that were silenced for CBP and p300 in absence and presence of TNF (Fig. 2a, d) as a basis to study individual functions of CBP and p300 in SF^14^ in regulating stress response pathways. In addition to our previously reported functions of CBP and p300 in regulating inflammatory pathways in SF, our pathway analysis of differentially expressed genes (DEG) in RNAseq data sets revealed an enrichment of target genes in a variety of stress response pathways (Fig. 2, Supplementary table 2). In line with p300 being the major writer of H3K27ac marks in SF^14^, the number of stress response pathways that we identified in SF silenced for p300 exceeded those identified in SF silenced for CBP. The majority of stress pathways regulated by CBP was associated with a prevention of DNA damage, such as “response to ionizing radiation”, “regulation of response to DNA damage stimulus”, “double-strand break repair”, “cellular response to UV, oxidative stress or chemical stress” (Fig. 2b, c; supplementary table 2). All of these pathways were additionally found to be regulated by p300. Among the pathways that were specifically regulated by p300 were the “response to hypoxia and oxygen levels”, as well as pathways associated with protein homeostasis, such as “autophagy”, “proteasomal pathways” and the response to “endoplasmatic reticulum (ER) stress” (Fig. 2e, f; supplementary table 2). To evaluate our key findings from the pathway analysis, we performed a set of confirmatory experiments and measured the expression of selected genes on RNA and protein levels, and evaluated functional consequences induced by DEG.

### The oxidative stress response is co-regulated by CBP and p300

**Figure 3:**
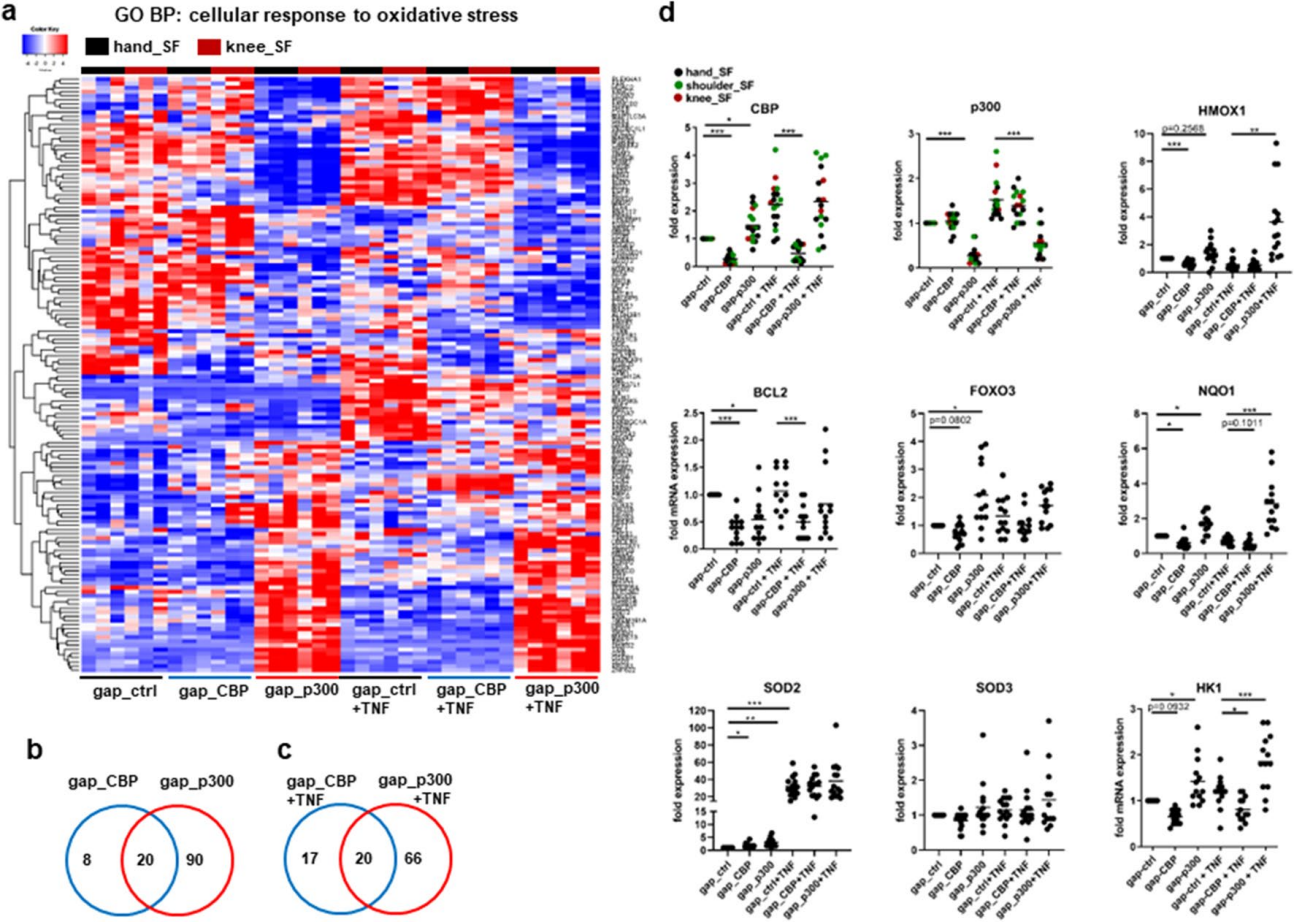
CBP and p300 regulate the response to oxidative stress. (**a**) Heatmap of DEG (± fold change > 1.5, FDR < 0.05) enriched in the biological process (BP) “cellular response to oxidative stress” that were identified by RNAseq of SF silenced for CBP or p300. Venn diagrams indicating the number of genes regulated by CBP and p300 in **(b)** unstimulated and **(c)** in TNF-stimulated SF. **(d)** Changes in mRNA expression for selected DEG were analyzed by Real-time PCR in an independent cohort of samples (n = 13–15) from data shown in (a). Results on mRNA expression of these in genes in SF from different joints (hand, shoulder, knee) are shown in supplementary Fig. 3. *p < 0.05, **p < 0.01, ***p < 0.005.

Oxidative stress response is an early event in the synovium of patients with RA^18^. We have identified the “cellular response to oxidative stress” as a shared stress pathway that was enriched in CBP and in p300 target genes in unstimulated and in TNF-stimulated SF (Fig. 3a). In both conditions, the majority of target genes in this pathway was regulated by p300, with 8.8% and 14.6% of genes being co-regulated by CBP (Fig. 3b, c). Among the 20 overlapping DEG identified in unstimulated SF were the apoptosis regulator BCL2, NAD(P)H quinone dehydrogenase 1 (NQO1), and the transcription factor forkhead box O3 (FOXO3). In TNF-stimulated SF, we identified additionally superoxide dismutase (SOD) 3 as a shared target gene of CBP and p300. We selected some DEG for validating the effects of CBP and p300 silencing on mRNA expression levels in an independent set of samples (Fig. 3d). Silencing reduced the expression of CBP mRNA by 73,6% (± 14,6%) and 78,9% (± 28,2%) in unstimulated and TNF-stimulated SF, respectively, and did not reduce the expression of p300. Silencing of p300 reduced the expression of p300 by 71,8% (± 18,1%) and 63,2% (± 30,2%), respectively, and did not reduce the expression of CBP (Fig. 3d). Silencing efficiency was similar in SF from hand, shoulder and knee SF. Similar effects of CBP and p300 silencing were only identified on the expression of BCL2 and SOD2. Silencing of CBP and p300 reduced the expression of BCL2 in unstimulated SF. In TNF-stimulated SF, p300 suppressed the expression of BCL2 only in knee SF (n = 3, supplementary Fig. 3. The expression of SOD2 was induced by silencing of CBP and p300 in unstimulated SF (Fig. 3d). CBP reduced the expression of SOD3 (p = 0.0674) only in SF from shoulders (n = 5) and knee (n = 3) but had no effect on hand SF (supplementary Fig. 3). We have detected opposing effects of CBP and p300 in regulating the expression of FOXO3, hexokinase (HK) 1, NQO1 and heme oxygenase 1 (HMOX1) (Fig. 3d). Whereas silencing of CBP suppressed the expression of these genes, silencing of p300 increased their expression. This suggests that CBP and p300 have opposing functions in regulating of oxidative stress response, at least in a subset of genes.

### CBP and p300 regulate autophagy

Our pathway analysis of DEG pointed to a role of p300 in the regulation of protein catabolism, including pathways associated with autophagy and the proteasome. Oxidative stress and hypoxia play a pivotal role in the pathogenesis of RA^18^ and are known inducers of autophagy^19^. We have identified p300 as a major factor that regulates not only oxidative stress response but also genes enriched in the “response to hypoxia” and “response to oxygen” levels (supplementary Fig. 4). Among the p300 target genes, were the metabolic enzymes HK2 and pyruvate dehydrogenase alpha 1 (PDHA1), the prolyl hydroxylase EGLN2, the pro-angiogenic factor VEGF, and the transcription factor peroxisome proliferator-activated receptor gamma (PPARG).

**Figure 4:**
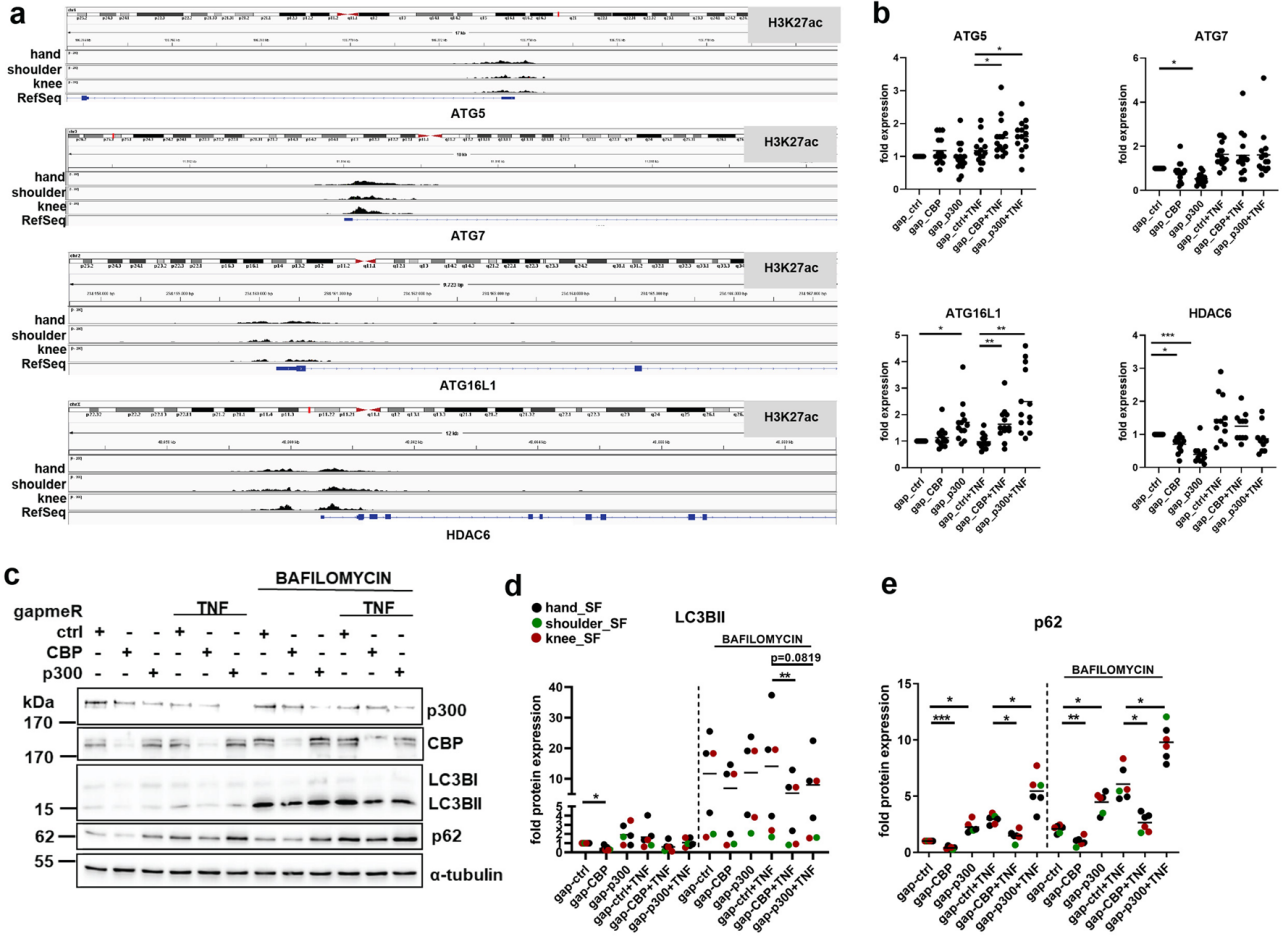
CBP and p300 regulate autophagy. (**a)** ChIPseq analysis of H3K27ac marks in the *ATG5*, *ATG7*, *ATG16L1* and *HDAC6* genetic loci up- and downstream of the transcription start sites in hand, shoulder and knee SF. **(b)** Changes in mRNA expression for selected autophagy-related genes were analyzed by Real-time PCR in an independent cohort (n = 12–15) of samples used for RNAseq. Results on mRNA expression of these in genes in SF from different joints (hand, shoulder, knee) are shown in supplementary Fig. 5. **(c)** Representative Western blots and densitometric analysis of **(d)**. LC3B (n = 6) and **(e)** p62 (n = 6). Markers of autophagy were analysed in absence and presence of bafilomycin A1 to assess the autophagix flux. Full length blots are shown in supplementary Fig. 6. *p < 0.05, **p < 0.01, ***p < 0.005.

In line with their HAT-dependent regulation, we have detected high levels of H3K27ac in the genomic regions surrounding the transcription start sites of autophagy related (ATG)5, ATG7, ATG16L1, and the autophagy regulator histone deacetylase 6 (HDAC6) (Fig. 4a). Silencing of p300 decreased basal levels of ATG7, and increased levels ATG5 in TNF-treated SF, as well as ATG16L1 levels in untreated and TNF-treated SF (Fig. 4b). CBP silencing had the same effect on ATG5 and ATG16L1 in TNF-treated SF, but did not affect their expression in unstimulated SF. Both silencing of CBP and p300 decreased the expression of HDAC6 in unstimulated SF. Upon TNF-stimulation, HDAC6 was only decreased by silencing of CBP or p300 in SF from shoulder and knee but not in SF from hand (supplementary Fig. 5). To functionally test potential changes in autophagy activity, we performed Western blotting using LC3B conversion and p62 protein expression as autophagy markers in presence and absence of the lysosomal inhibitor bafilomycin A1 (Fig. 4c–e, supplementary Fig. 6). Silencing of CBP reduced the conversion of LC3B and the protein expression of p62 in presence and absence of TNF. Results were similar in presence of bafilomycin A1, indicating a decrease in autophagosome synthesis. In contrast, the conversion of LC3B and p62 expression were increased after silencing of p300 in unstimulated SF, indicating increased autophagy. This effect was lost for LC3B after treatment with TNF, and LC3B conversion was even decreased in presence of bafilomycin A1. This indicates a late-stage block of autophagy after silencing of p300 in TNF-stimulated SF.

### p300 silencing affects proteasome activity

**Figure 5:**
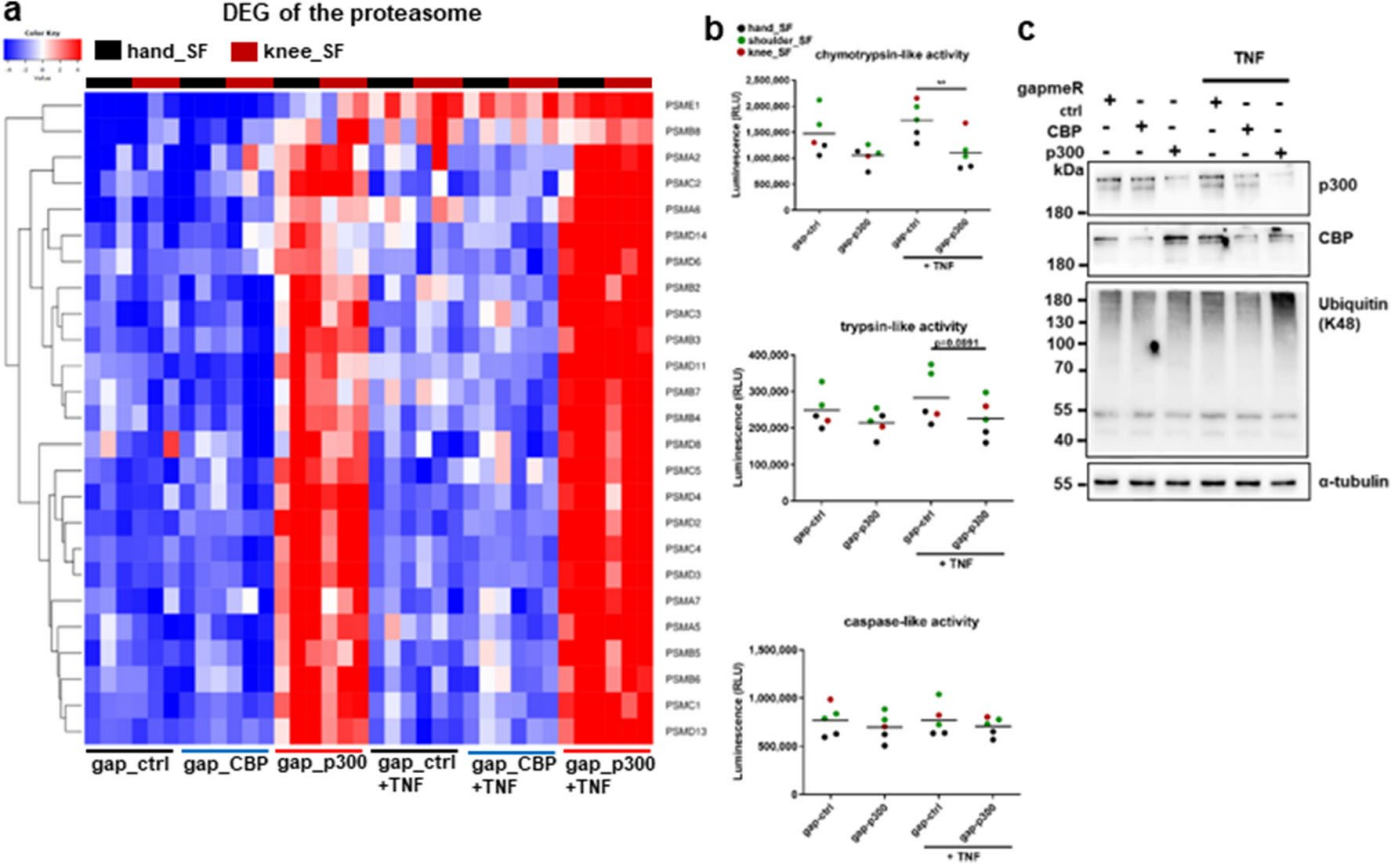
p300 regulates the expression and activity of proteasomal components. (**a**) Heatmap of DEG (± fold change > 1.5, FDR < 0.05) encoding components of the proteasome, and selected from the enriched biological processes (BP) “proteasomal protein catabolic process” and “proteasome-mediated ubiquitin-dependent protein catabolic process”. **(b)** Chymotrypsin-like, trypsin-like and caspase-like proteasome activities in SF (n = 5) silenced for p300 in absence and presence of TNF. **(c)** The accumulation of poly-ubiquitinated proteins in SF silenced for CBP and p300, respectively, was analysed by Western blotting. A representative Western blot is shown (n = 4). Full length blots are shown in supplementary Fig. 7 **p < 0.01.

Our pathway analysis of DEG suggested a pivotal and specific role of p300 in regulating proteasome function (supplementary table 2). We have detected an increased expression of the majority of genes encoding for proteasome machinery components after silencing of p300 (Fig. 5a), which were enriched in the pathways “proteasomal protein catabolic process” and “proteasome-mediated ubiquitin-dependent protein catabolic process”. Silencing of CBP had no effect on the expression of these genes (Fig. 5a). Our functional analysis of the proteasome enzymatic activities revealed that silencing of p300 suppressed the chymotrypsin-like and trypsin-like activities, whereas the caspase-like enzymatic activity was not affected (Fig. 5b). These data suggest that the increased expression of proteasome-related genes on mRNA levels was compensatory for the decreased enzymatic activities after silencing of p300. In line with decreased enzymatic proteosome activities, we have detected an increased accumulation of poly-ubiquitinated proteins in SF that were silenced for p300, in particular in samples that were stimulated with TNF. This was effect was not detected in SF after silencing of CBP (Fig. 5c).

## Discussion

Hypoxia, the formation of reactive oxygen species (ROS), and subsequent oxidative stress in synovial tissues are key events in the pathogenesis of RA^1^. We provide here evidence that CBP and p300 are pivotal regulators of the adaptive response of SF that integrate the cell`s transcriptional and functional regulation of stress response pathways.

CBP and p300 are HATs and writers of H3K27ac marks, activating post-translational histone modifications present in enhancers and promoters^20^. Among the CBP/ p300 target proteins are beside histones several non-histone proteins, including many transcription factors and signaling effectors^16,21^. Despite their high protein sequence homology, CBP and p300 have several individual functions, identified by our own and other studies^12–14^.

In SF, a sustained H3K27ac in inflammatory gene promoters was associated with a prolonged and persistent expression of the corresponding genes^22^. We have recently shown that p300 is the major HAT in SF that exerts pro- and anti-inflammatory roles. This is contrast to CBP, that exerted anti-inflammatory effects upon silencing, and specifically regulated TNF-induced interferon signature gene expression^14^. Although we have ruled out that silencing of p300 additionally decreased CBP and vice versa, we cannot completely rule out other potential off target effects following our silencing approach. *Krosel* et *al.* have shown that inhibitors targeting the HAT or bromodomain of CBP/ p300 to a large extent resemble effects of p300 silencing, including an increased expression of TNF-induced pro-inflammatory gene expression^14^.

Our RNAseq data provides evidence that numbers of p300-regulated target genes exceed those of CBP-regulated target genes also in terms of stress response. Whereas the cellular response to oxidative stress and autophagy were co-regulated by CBP and p300, genes associated with a response to oxygen levels, hypoxia and pathways associated with proteasome regulation and function were specifically enriched after silencing of p300. In pathways that were co-regulated by CBP and p300, we have identified several genes that were regulated in opposite directions. These results point to individual functions of the two enzymes on target genes levels, similarly to what we have already observed for many inflammatory genes^14^. In addition, a small number of measured CBP and p300 target genes, namely BCL2, SOD3, and HDAC6, might be regulated in a joint-specific manner, as indicated by our Real-time PCR results in a limited number of samples for each joint location. *Frank-Bertoncelj* et al. have previously shown that H3K27ac is one of the mechanisms controlling the joint-specific expression of homeobox (HOX) transcription factors in SF from different locations^7^. To draw a final conclusion whether stress-associated target genes are regulated in a joint-specific manner, larger numbers of SF from different joints would be needed, along with H3K27ac ChIPseq data in unstimulated and TNF-stimulated SF from different joints.

**Figure 6:**
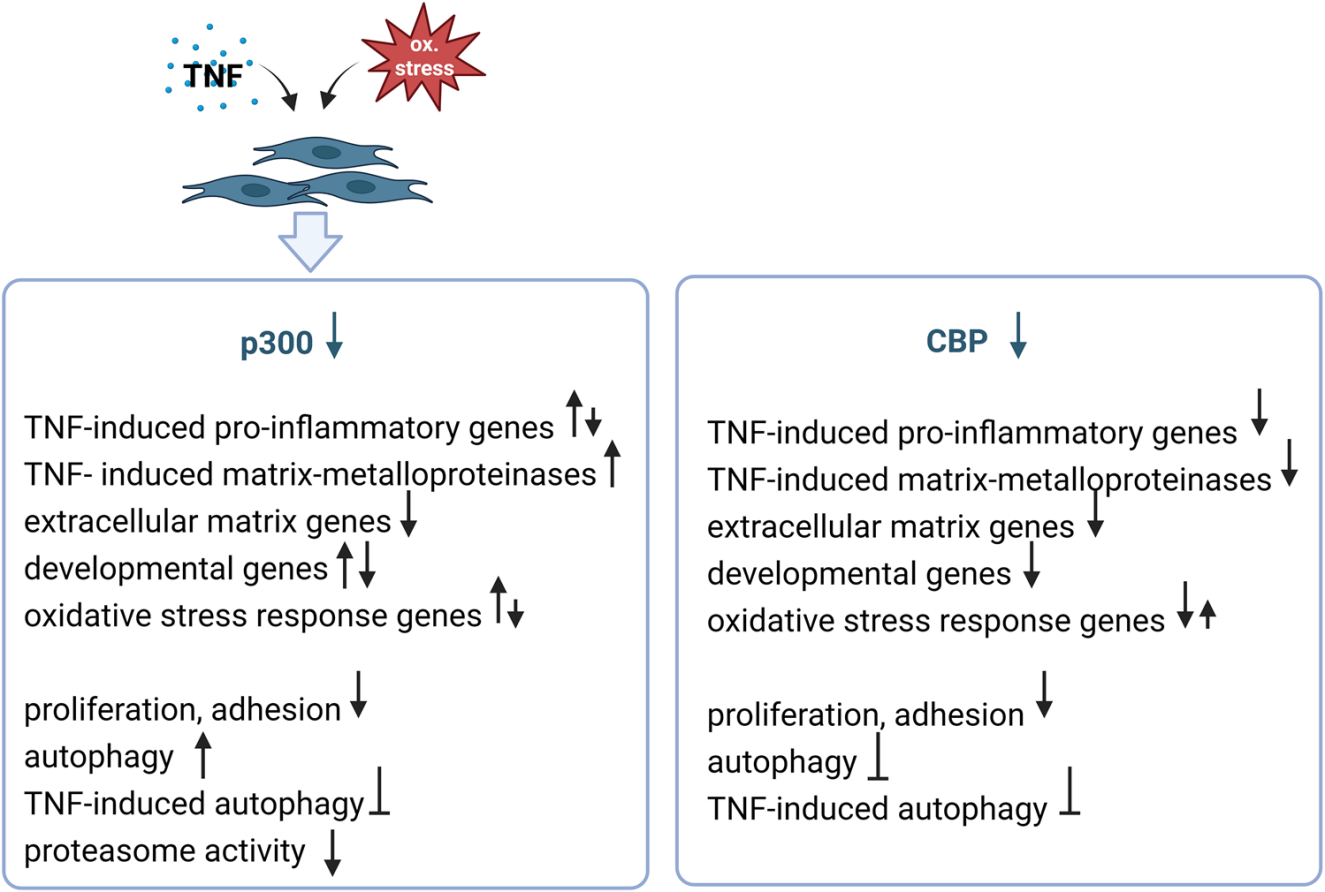
Summary of CBP- and p300-regulated pathways in SF. The expression of p300 but not of CBP is down regulated in synovial fibroblasts upon exposure to TNF and oxidative stress. Effects of p300 and CBP silencing are shown based on findings from this and a previous study^14^. Down facing arrows indicate suppressed expression or function, up facing arrows indicate increased expression or function. The figure was created by BioRender.com.

In addition to different roles of CBP and p300 in regulating target gene expression, we showed here a differential regulation of CBP and p300 by stimulating SF with 4-HNE and TNF (Fig. 6). These factors are present in the synovial microenvironment in RA, and mimic the oxidative stress and inflammation, respectively. Whereas 4-HNE and TNF, similarly to H_2_O_2_, suppressed the expression of p300, CBP was not affected. 4-HNE is a lipid peroxidation product generated upon increased levels of ROS. Levels of 4-HNE are elevated in serum, synovial fluids and synovial tissues of RA patients, and serum levels of 4-HNE correlate with the structural damage such as erosions in the early stage of RA^23,24^. As mimicked by our silencing approach, the TNF- and 4-HNE-mediated suppression of p300 expression in the synovial RA microenvironment has fundamental consequences on SF behaviour. Our data sets from the previous^14^ and the current study indicate that a decreased expression of p300 was associated with an increased expression of many inflammatory cytokines, chemokines matrix metalloproteinases, and stress response genes in SF. Among these genes were HK2, a marker indicting the metabolic switch of SF towards glycolysis, and VEGF, a pro-angiogenic factor secreted to overcome hypoxia^1^.

TNF stimulation of SF was shown to induce markers of endoplasmatic reticulum (ER) stress and autophagy^25^. Our data suggest that CBP and p300 regulate autophagy at transcriptional level and effects autophagic flux. The assessment of autophagy in presence of the lysosomal inhibitor bafilomycin A1 indicated that CBP and p300 regulate autophagy function at different stages within the autophagic process. CBP silencing affected autophagosome synthesis. In contrast, silencing of p300 induced autophagy in unstimulated SF, and induced a late-stage block of autophagy in TNF-stimulated SF, a condition in which poly-ubiquitinated proteins in SF accumulated. Accordingly, silencing of p300 only increased cell death in presence of TNF, as indicated previously^14^. *Kato* et *al*. have previously shown that autophagy induction partially compensated for an impaired clearance of poly-ubiquitinated proteins in SF after blocking proteasome function, pointing to a protective effect of autophagy induction in SF in such conditions^5^. Here we observed a similar compensatory mechanism after silencing of p300, which was associated with a suppression of proteasome enzymatic activities and an induction of autophagy. This finding is in line with a previous study in HeLa cells in which the knockdown of p300 was associated with a decreased acetylation of autophagy-related proteins and increased levels of autophagy^26^.

Acetylation and deacetylation of components of the autophagy machinery control all steps of this catabolic process from autophagosome initiation to LC3 conjugation, cargo assembly and autophagosome-lysosome fusion^27,28^. Different classes of acetyltransferases, including CBP and p300, and deacetylases, including sirtuin1, HDAC4 and HDAC6 are involved in the regulation of autophagy^27,29^. In addition, the function of autophagy-related transcription factors, such as transcription factor EB (TFEB), Foxo1 and Foxo3, are regulated by deacetylation^28,30^. The increased translation of FOXO3 mRNA has recently been described to facilitate autophagy initiation^31^. We showed here that in unstimulated SF, FOXO3 mRNA was increased upon silencing of p300, a condition in which autophagic flux was increased. HDAC6 binds to poly-ubiquitinated proteins in SF^29^, and promotes autophagy by facilitating autophagosome-lysosome fusion^27^. On the other hand, HDAC6 was also shown to suppress autophagy by deacetylating TFEB and Foxo1^30^. This might explain the inverse regulation of HDAC6 and ATG5 and ATG16L1 in SF.

A global analysis of the CBP/ p300-dependent acetylome in mouse embryonic fibroblasts (MEF) suggested that also proteasome functions might be regulated by these enzymes^21^. The majority of components of the proteasome machinery exhibited numerous CBP/ p300-dependent acetylation sites in regulatory and enzymatic subunits in MEF (http://p300db.choudharylab.org). Furthermore, ATG5 and ATG16L1, two proteins essential for the assembly of autophagosomes, exhibited CBP/ p300-dependent acetylation sites^21^. Whether proteasome and autophagy components are acetylated in a CBP- and p300-dependent manner in SF, remains to be functionally evaluated^21^. Since CBP/ p300-dependent acetylation sites in MEF were analyzed after a combinatorial knockout of both enzymes, it is not clear which of them is the major enzyme in regulating the post-translational acetylation of proteins involved in the regulation of autophagy and proteasome activities.

In summary, we have identified CBP and in particular p300 as pivotal regulators of stress response pathways in SF, with overlapping and distinct functions within specific pathways.. The downregulation of p300 by TNF and oxidative stress provides a mechanism underlying SF activation in the synovial microenvironment.

## Material and methods

### Patient samples and cell preparation

Synovial tissue specimens were obtained from hand, shoulder and knee joints of RA patients undergoing joint replacement surgery (Schulthess Clinic Zurich, Switzerland)^32^. All patients fulfilled the criteria for the classification of RA^33^. SF were isolated and cultured as described elsewhere^7^ and used between passages four and eight for all experiments. The study was conducted in accordance with the Declaration of Helsinki, and approved by the Cantonal Ethics Committee Zurich, Switzerland (approval numbers 2019–00,115 and 2019–00,674)^32^. Informed consent was obtained from all patients prior the inclusion into the study.

### Silencing of CBP and p300

SF were transfected with antisense LNA gapmeRs targeting CBP, p300 or gapmeR control (Qiagen) using lipofectamine (Thermo Scientific) as described previously^14^. Twenty-four hours after transfection, cells were stimulated with TNF (10 ng/ml) for 24 h. Knockdown of CBP and p300 was verified by real-time PCR and Western blotting^14^.

### RNA sequencing of SF

We have performed RNA sequencing (RNAseq) of SF (n = 6, with 3 cultures derived from hand and 3 from knee) silenced for CBP, p300 or controls in absence and presence of TNF (10 ng/ml). Experimental details on RNAseq have been published in our previous study^14^. RNAseq data have been deposited in NCBI`s Gene Expression Omnibus^34^ and are accessible through the GEO Series accession number GSE236122. Differentially expressed genes (DEG) entered pathway enrichment analysis using Gene Ontology (GO) terms and the “clusterProfiler" package of Bioconductor. Stress related pathways were selected by expert opinion to prepare customized graphs.

### Real-time PCR

Total RNA was isolated using the RNeasy Mini Kit (Qiagen) including on-column DNaseI digest and was reversed transcribed^32^. Real-time PCR (7900HT real-time PCR system, Life Technologies) was performed using self-designed primers (Microsynth, supplementary table 1) and SYBR green (Roche)^35^. Dissociation curves and samples containing the untranscribed RNA were measured in parallel. Constitutively expressed human ribosomal protein large P0 (RPLP0) was measured for internal standard sample normalization and relative mRNA expression levels were calculated by the comparative threshold cycle method (ΔΔCt)^36^.

### Treatment of SF

For assessing effects on autophagy, SF were silenced for CBP or p300 and treated with TNF as described above. Bafilomycin A1 (100 nM, Tocris Bioscience) was added for 4 h prior to harvesting the cells. For assessing the regulation of CBP and p300 protein expression, SF were treated with TNF (10 ng/ml), 4-hydroxynonenal (4-HNE, 5 µM, Sigma Aldrich), or a combination of both for 48 h, or with H_2_O_2_ (0,1 and 0,3 mM, Sigma Aldrich) for 24 h. Controls were treated with matched amounts of ethanol.

### Western blotting

SF were lysed in Laemmli buffer (62.5 mM TrisHCl, 2% SDS, 10% glycerol, 0.1% bromphenolblue, 5 mM β-mercaptoethanol). Western blotting was performed as described previously^32^. In brief, whole cell lysates were separated on SDS polyacrylamide gels and electro blotted onto nitrocellulose membranes (Whatman). Membranes were blocked for 1 h in 5% (w/v) non-fat milk in TBS-T (20 mM Tris base, 137 mM sodium chloride, 0.1% Tween 20, pH 7.6). Membranes were cut in pieces covering different protein sizes prior hybridization with primary antibodies, enabling us to use the same membrane for detection of different proteins without stripping of the membranes. The membranes were probed with antibodies against CBP (Cell Signaling), p300 (abcam), light chain 3 B (LC3B; Cell Signaling), p62 (abcam), ubiquitin (Lys48; Millipore) or α-tubulin (abcam). We have tested the specificity of all antibodies previously^5,14,29^. As secondary antibodies, horseradish peroxidase-conjugated goat anti-rabbit or goat anti-mouse antibodies (Jackson ImmunoResearch) were used. Signals were detected using the ECL Western blotting detection reagents (GE Healthcare) and the Alpha Imager Software system (Alpha Innotech). Band intensities were measured using the Alpha Imager Software, and band intensities were calculated relative to the expression of α-tubulin.

### ELISA

VEGF was measured in cell culture supernatants using ELISA according to the manufacturer’s instructions (R&D Systems).

### Proteasome activity assay

SF were transfected and treated as described above. Proteasome activities were measured using the Proteasome-Glo 3-Substrate Cell Based Assay System (Promega) following the manufacturer`s instructions.

### Analysis of ChIPseq data sets

ChIPseq data sets for H3K27ac in hand, shoulder and knee SF were generated previously^7^ and are available at the GEO repository accession GSE163548^37^. The presence of H3K27ac in genomic regions autophagy-related genes was visualized using the Integrative Genomics Viewer (IGV)^38^.

### Statistical analysis

Statistical analysis on data sets was carried out by using the GraphPad Prism Software as described previously^32^. N numbers in all experiments represent biological samples from different patients. Differences between experimental groups were analyzed by analysis of variance (ANOVA) followed by Tukey`s multiple comparison test. Data that were not normally distributed, were analyzed by Friedmann test followed by the post hoc Dunn`s multiple comparison test. Data are reported as means ± standard deviations. P values < 0.05 were considered significant.

### Supplementary Information

Supplementary Information.

## Data Availability

The RNAseq data discussed in this publication have been deposited in NCBI`s Gene Expression Omnibus^34^ and are accessible through the GEO Series accession number GSE236122 (https://www.ncbi.nlm.nih.gov/geo/query/acc.cgi?acc=GSE236122). Other datasets used and/or analysed during the current study are available from the corresponding author on reasonable request.
