# Supplementary material for: The histone acetyl transferases CBP and p300 regulate stress response pathways in synovial fibroblasts at transcriptional and functional levels
Source: Sci Rep. 2023 Oct 10;13:17112. doi: 10.1038/s41598-023-44412-z (PMC10564874; doi:10.1038/s41598-023-44412-z)
Supplement: Supplementary file 1 — Supplementary Information. [file 41598_2023_44412_MOESM1_ESM.pdf]

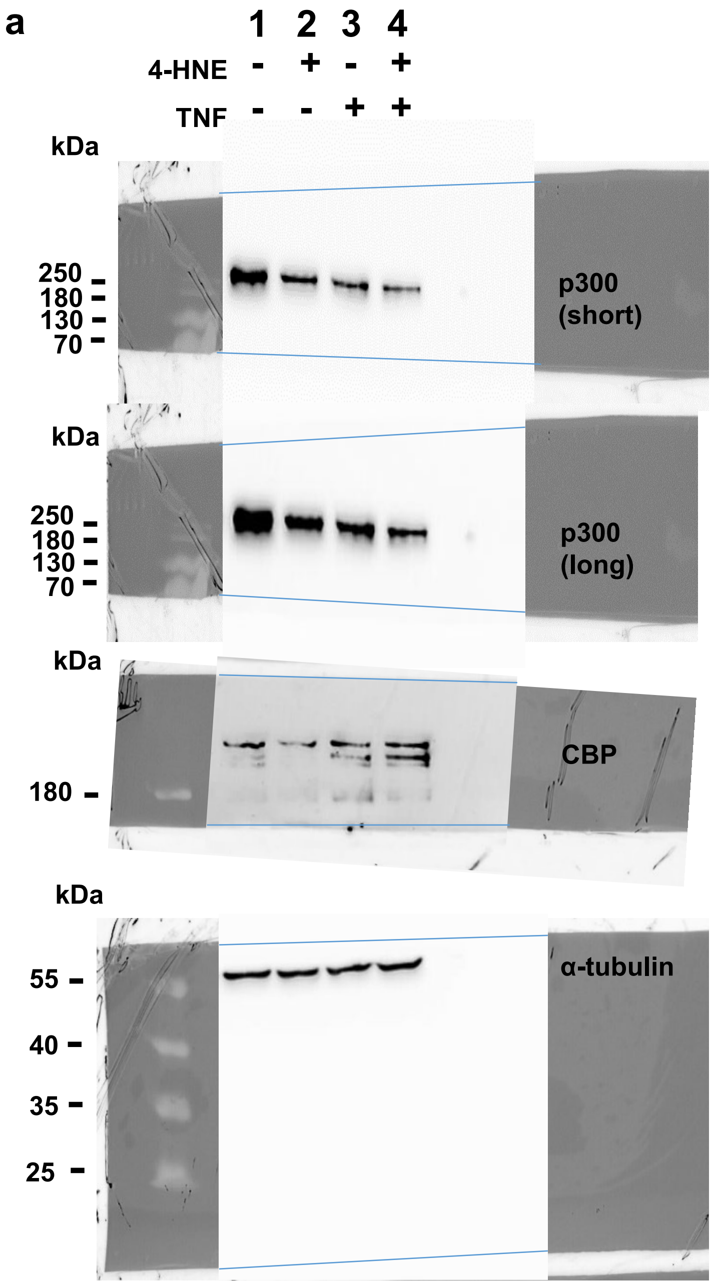

Supplementary Figure 1: Regulation of CBP and p300 protein expression. Full length blots of Western blots shown in Figure 1. SF were treated with 4-HNE and TNF, or a combination of both. Membranes were cut prior probing with the primary antibodies. Protein size markers are shown on the left side of membranes. **(a)** The original size of the membranes is shown in the background of the Western blot images. The membrane edges are indicated by blue lines. Original Western blots for **(b)** CBP, **(c)** p300, and **(d)** α-tubulin from all replicates shown in Figure 1. Labelling of samples 1-4 is according to conditions shown in (**a**). Black boxes hide bands from samples harvested at other time points that were not further discussed in the manuscript.

b

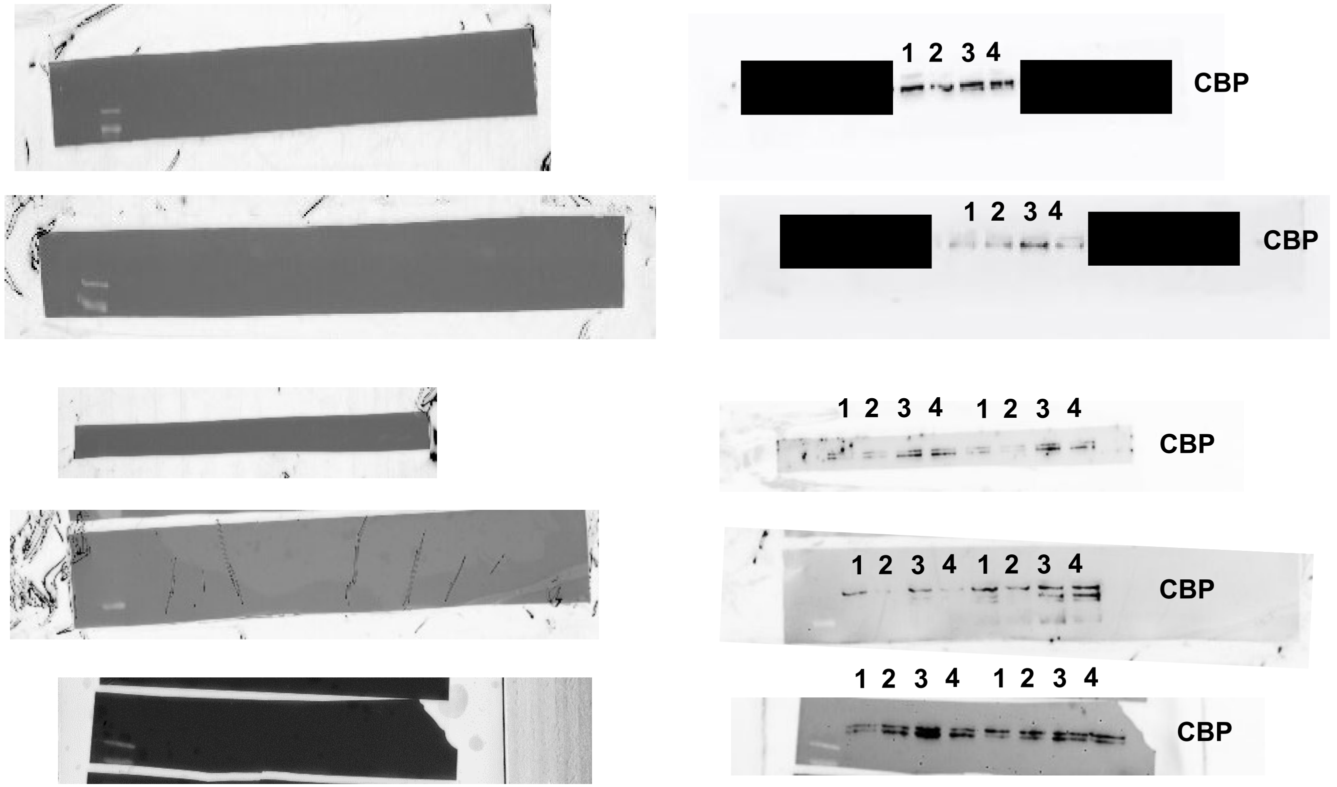

c

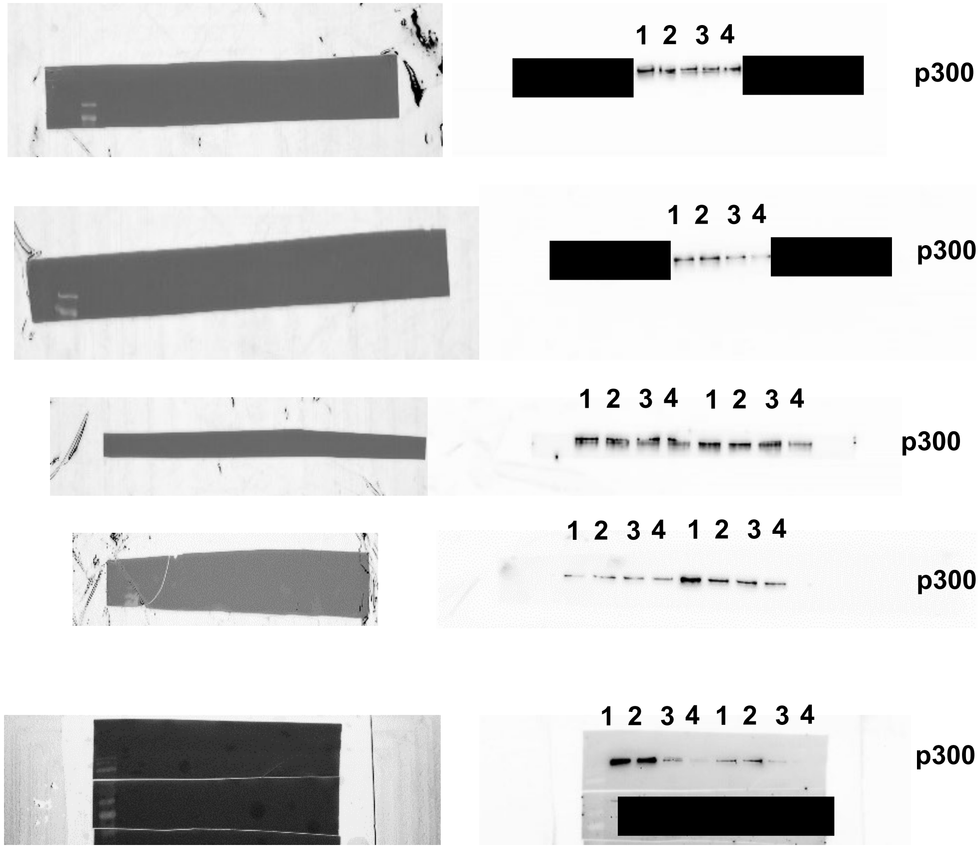
1 2 3 4
p300
1 2 3 4
p300
1 2 3 4 1 2 3 4
p300
1 2 3 4 1 2 3 4
p300
1 2 3 4 1 2 3 4
p300

d

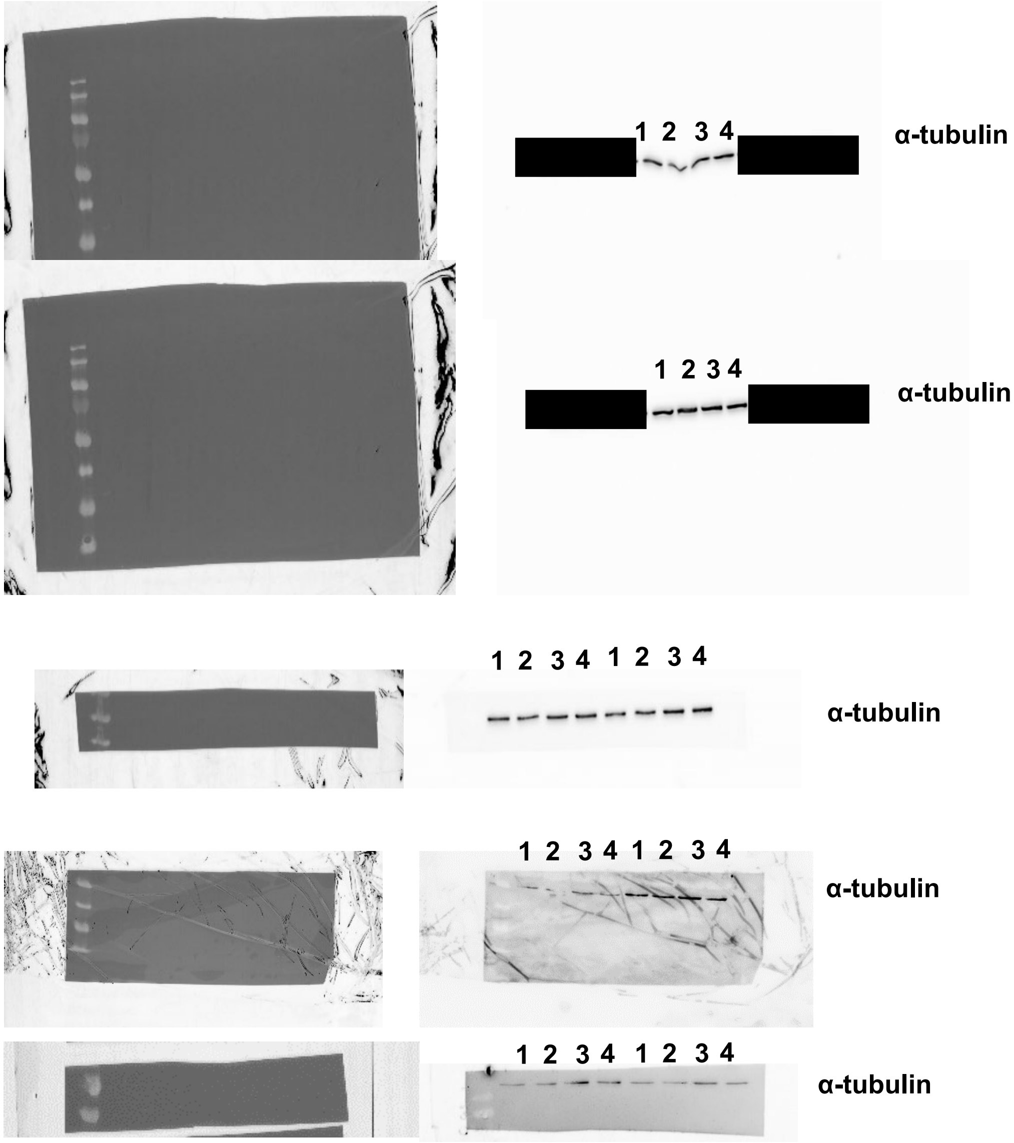
1 2 3 4
α-tubulin
1 2 3 4
α-tubulin
1 2 3 4 1 2 3 4
α-tubulin
1 2 3 4 1 2 3 4
α-tubulin
1 2 3 4 1 2 3 4
α-tubulin

a

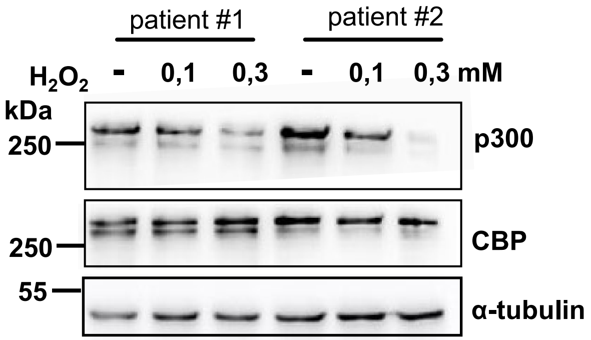

b

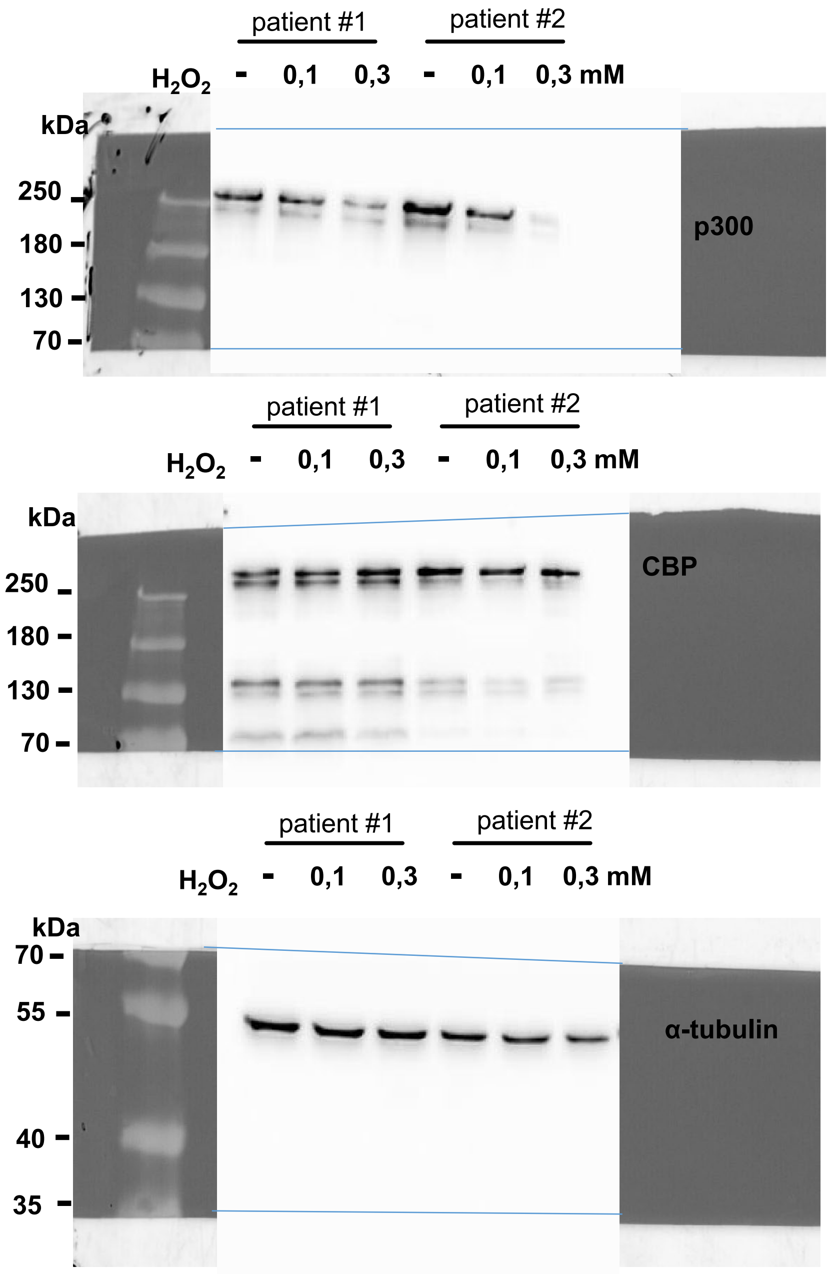

Supplementary Figure 2: Regulation of CBP and p300 protein expression by H_2O_2. (a) Western blots of SF (n=2) that were treated with 0,1 or 0,3 mM H_2O_2 for 24h. **(b)** Full length blots of Western blots shown in (a). Membranes were cut prior probing with the primary antibodies. Protein size markers are shown on the left side of membranes. The original size of the membranes is shown in the background of the Western blot images. The membrane edges are indicated by blue lines.

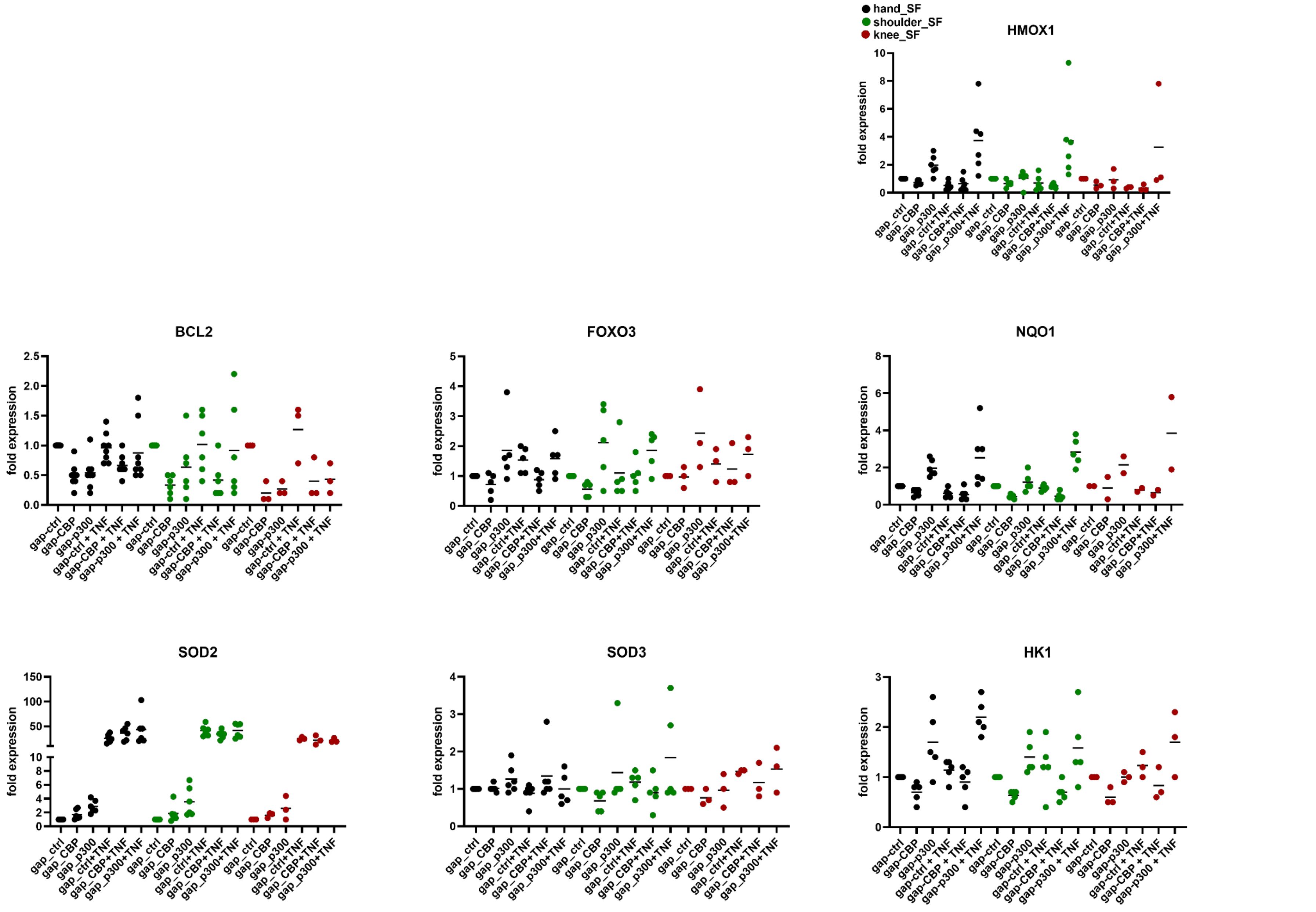

Supplementary Figure 3: CBP and p300 regulate the response to oxidative stress. Changes in mRNA expression for selected DEG were analyzed by Real-time PCR in SF from hand (black), shoulder (green) and knee (red). Statistical analysis of the cumulative results is shown in Figure 3.

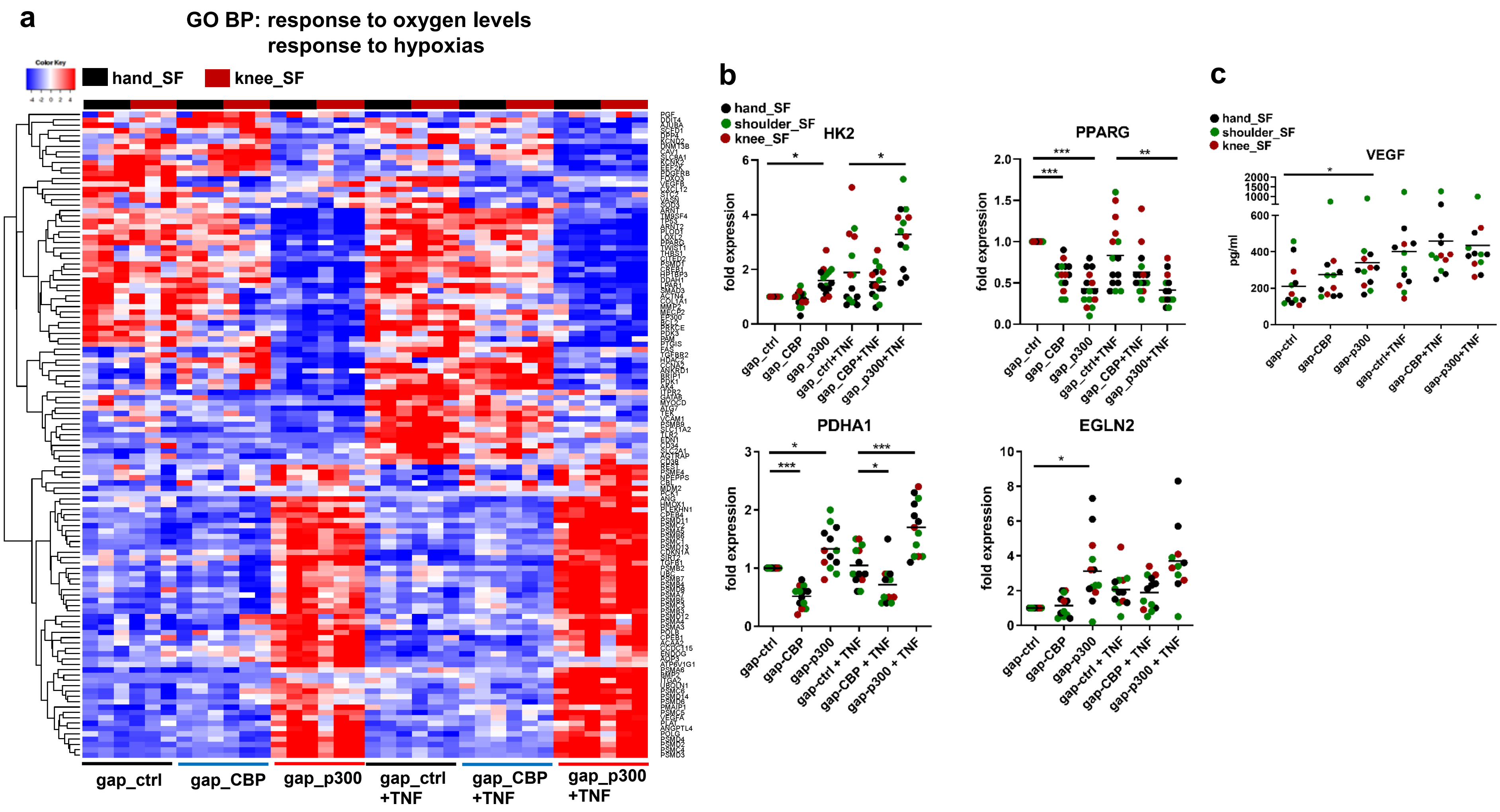

Supplementary Figure 4: : p300 regulates the response to oxygen levels and hypoxia. (a) Heatmap of DEG (±fold change > 1.5, FDR <0.05) enriched in the biological processes (BP) "response to oxygen levels" and "hypoxia" that were identified by RNAseq of SF silenced for p300. (b) Changes in mRNA expression for selected DEG were analyzed by Real-time PCR in an independent cohort of samples (n=13-15) from data shown in (a). (c) The secretion of VEGF was measured by ELISA. p<0.05, **p<0.01, ***p<0.005

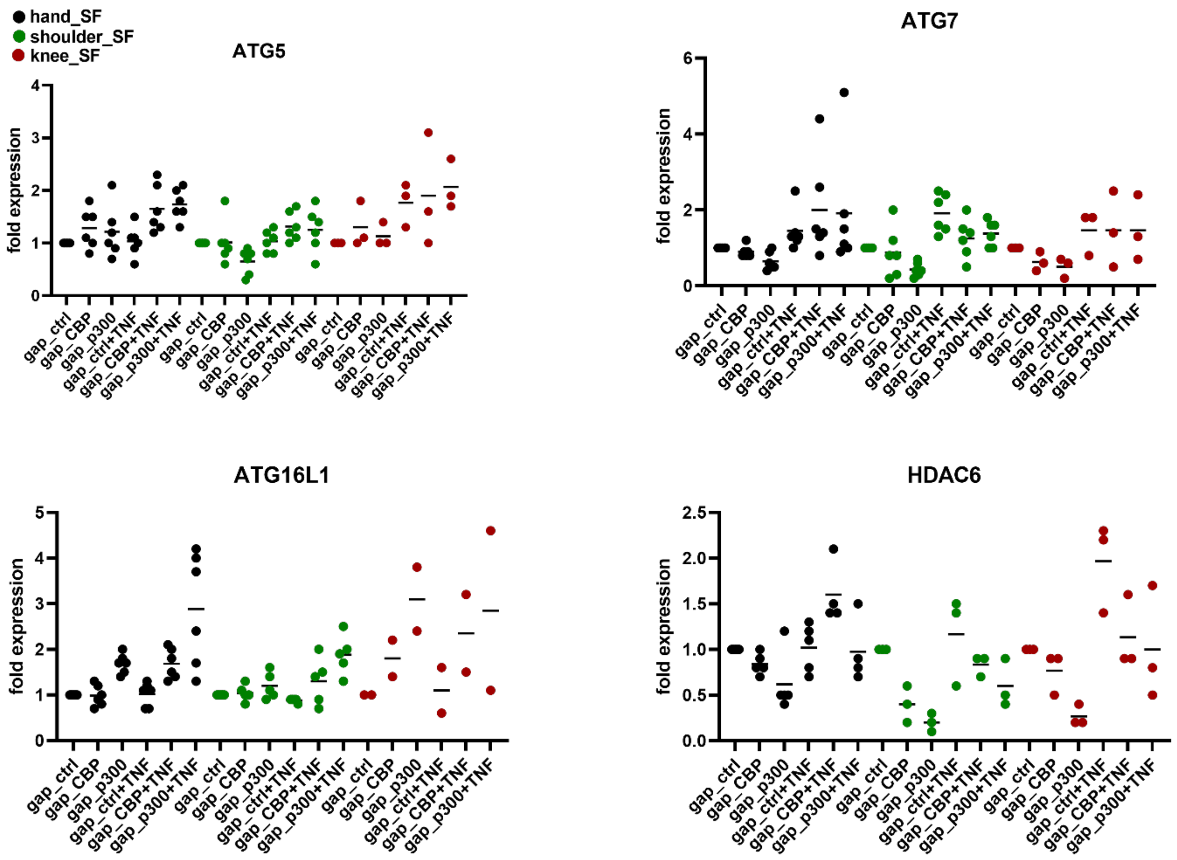

Supplementary Figure 5: CBP and p300 regulate autophagy-related genes. Changes in mRNA expression for selected DEG were analyzed by Real-time PCR in SF from hand (black), shoulder (green) and knee (red). Statistical analysis of the cumulative results is shown in Figure 4.

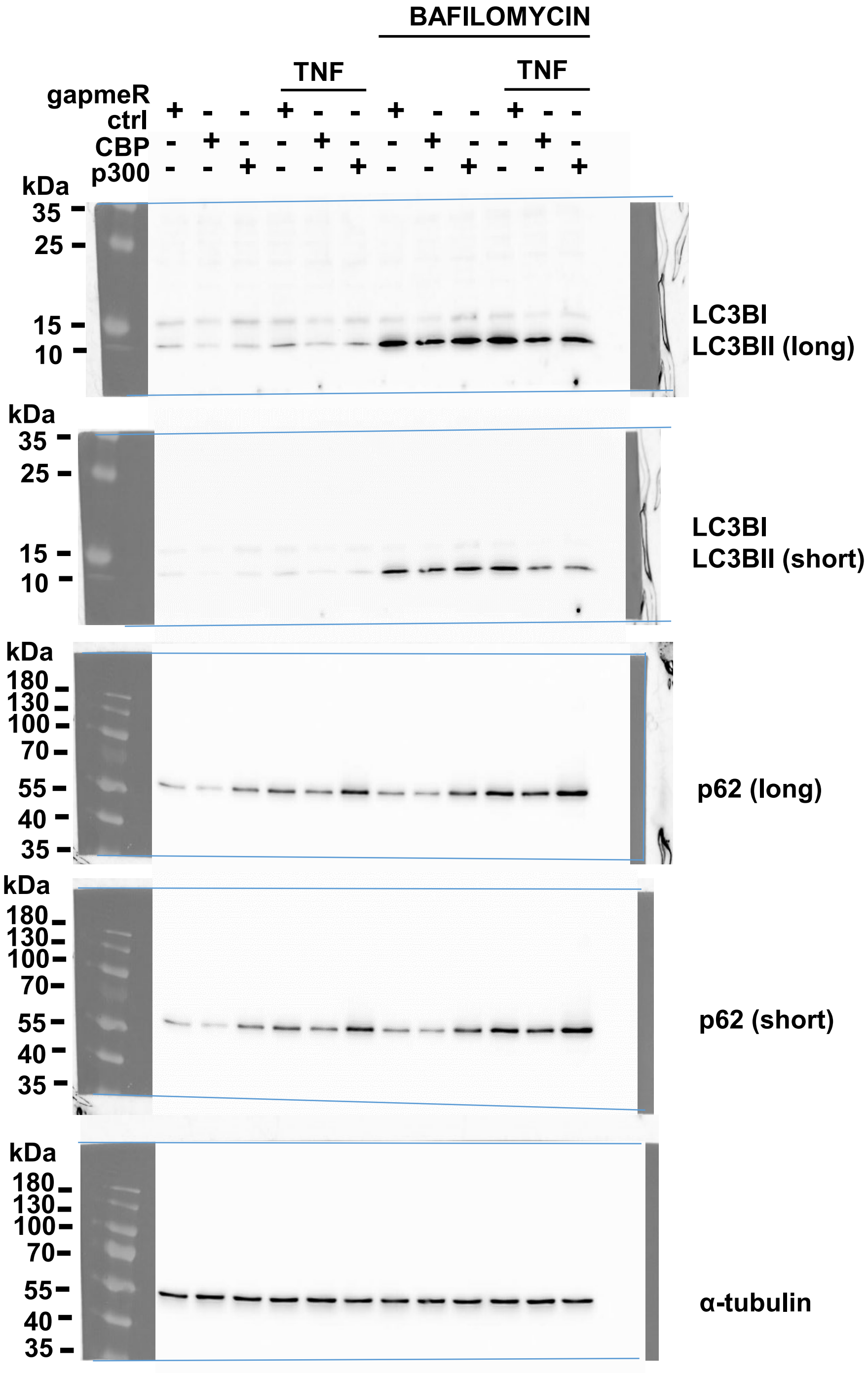

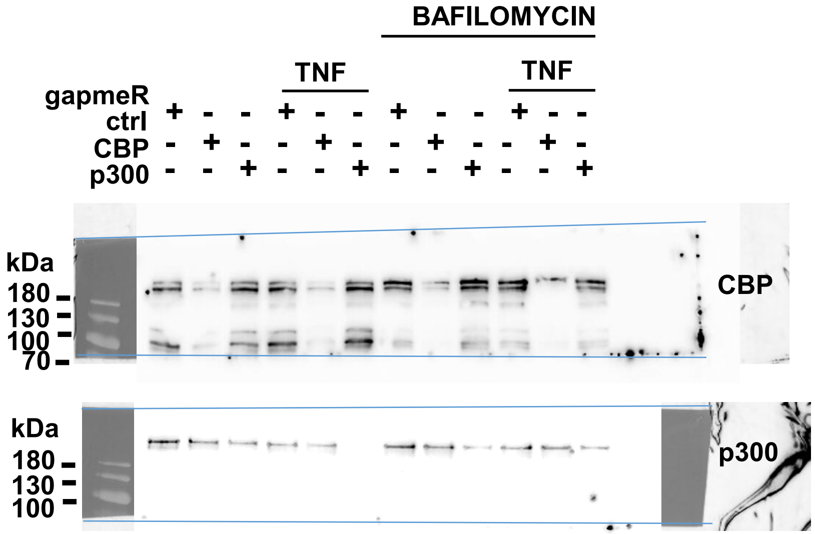

Supplementary Figure 6: CBP and p300 regulate autophagy. Full length blots of Western blots shown in Figure 5. The conversion of LC3B and p62, markers of autophagy, were analysed in absence and presence of bafilomycin A1 to assess the autophagix flux. Membranes were cut prior probing with the primary antibodies. Protein size markers are shown on the left side of membranes. The original size of the membranes is shown in the background of the Western blot images. The membrane edges are indicated by blue lines.

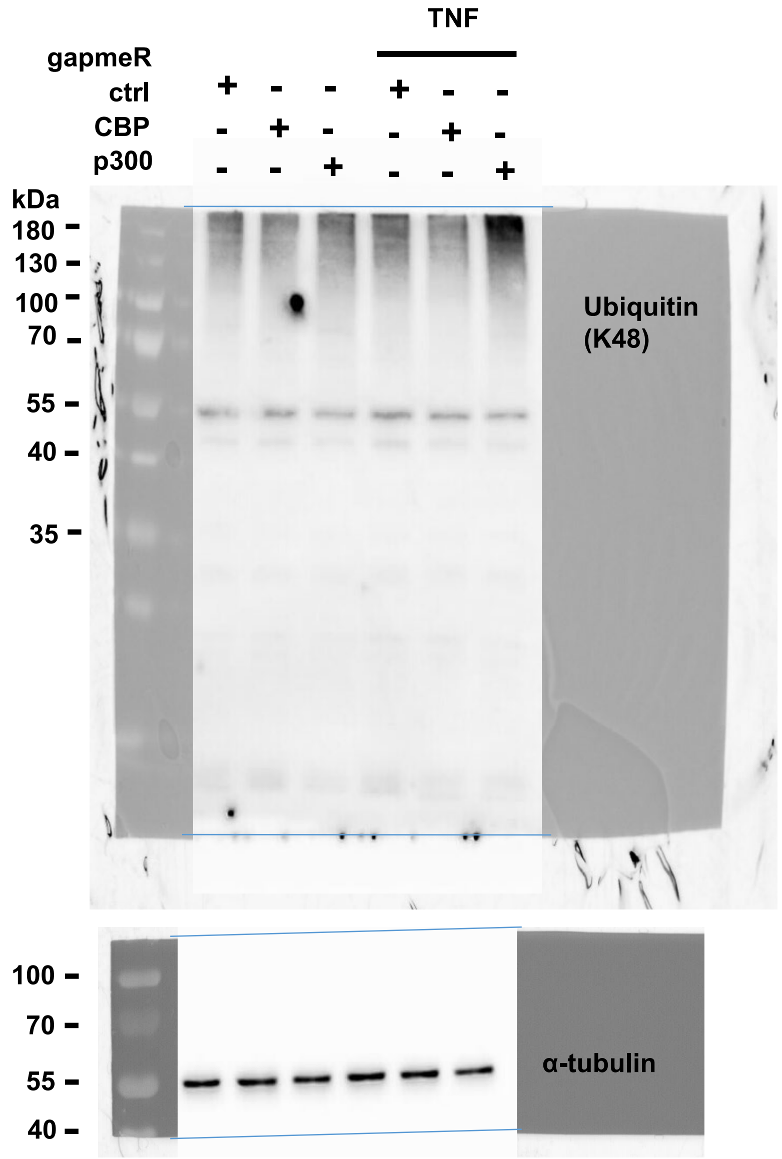

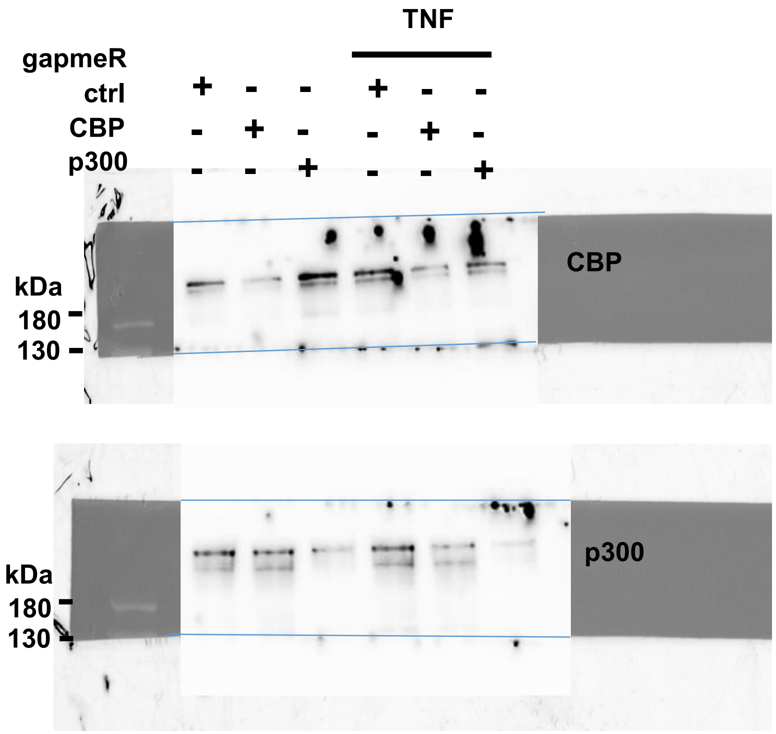

Supplementary Figure 7: p300 regulates the turnover of poly-ubiquitinated proteins. Full length blots of Western blots shown in Figure 6. The accumulation of polyubiquitinated proteins was analysed in SF after silencing of CBP or p300 and stimulation with TNF. Membranes were cut prior probing with the primary antibodies. Protein size markers are shown on the left side of membranes. The original size of the membranes is shown in the background of the Western blot images. The membrane edges are indicated by blue lines.

	Sequence (5`- 3`)
CBP fw	GCAAGCAAACGGAGAGGTTC
CBP rev	ATGCACAATGGGCAACTTGG
p300 fw	CAGGCATGGTTCCAGTTTCC
p300 rev	CAGGTAGAGGGCCATTAGAAGTC
HMOX1 fw	AGACGGCTTCAAGCTGGTGAT
HMOX1 rev	CCTTGTTGCGCTCAATCTCCT
HK2 fw	TGCCACCAGACTAAACTAGACG
HK2 rev	CCCGTGCCCACAATGAGAC
NQO1 fw	TGGCTAGGTATCATTCAACTC
NQO1 rev	CCTTAGGGCAGGTAGATTCAG
SOD3 fw	GACAGCCTGCGTTCCTGG
SOD3 rev	CTGGAGTCGGGCACCTTTC
ATG7 fw	TAGCCCAGATTGTCCTAAAGCAGTTGG
ATG7 rev	GATGACTCAGCTAACCTTTTAGGGTCC
SOD2 fw	CGGGGACACTTACAAATTGC
SOD2 rev	CCTTGCAGTGGATCCTGATT
ATG5 fw	AATCGGAAACTCATGGAATATCC
ATG5 rev	CGAAACAGCTTCTGAATGAAAGG
PPARg fw	ATGCCTTGCAGTGGGGATGTCTCAT
PPARg rev	CATCCGCCCAAACCTGATGGCATTA
HDAC6 fw	GAAAGTCACCTCGGCATCAT
HDAC6 rev	TAGTCTGGCCTGGAGTGGAC
BCL2 fw	AGGATTGTGGCCTTCTTTGA
BCL2 rev	ACGCTCTCCACACACATGAC
ATG16L1 fw	GCTCTCTGTATATCTGGAGTG
ATG16L1 rev	CCACAGCACAGCTTTGCATC
HK1 fw	GCGTGATAACAAGGGCACAC
HK1 rev	GGAATACTGTGGGTGCGTCT
EGLN2 fw	TCCTGTCTCCGGCCACC
EGLN2 rev	GAGCTCTTCTCGTGCCCTG
FOXO3 fw	CTACGAGTGGATGGTGCGTT
FOXO3 rev	TGTGCCGGATGGAGTTCTTC
RPLP0 fw	GCGTCCTCGTGGAAGTGACATCG
RPLP0 rev	TCAGGGATTGCCACGCAGGG

Supplementary table 1: Sequences of primers used for Real-time PCR

Supplementary table 2: Differentially expressed genes (± fold change > 1.5, FDR <0.05) of RNAseq data of SF silenced for CBP and p300, respectively, entered pathway enrichment analysis for Gene Ontology (GO) biological process (BP). Details on pathways shown in figure 1 are presented.

ID	Description	GeneRatio	pvalue	p.adjust	qvalue	geneID
**CBP vs control**						
GO:0006302	double-strand break repair	33/890	9,54E-08	7,74E-05	7,38E-05	MMS19/NSMCE1/MCM3/ERCC1/MCM4/CHEK1/H4C3/NUDT16L1/MCM6/MCM2/DCLRE1B/TNKS1BP1/SLF1/SPIRE2/MCM7/BRCC3/FIGNL1/RFWD3/RAD51/BLM/RAD51AP1/GEN1/POLN/ATP23/CDCA5/CHEK2/MSH2/TRIP13/BRCA2/CDC7/XRCC2/TONSL/H4C13
GO:0031670	cellular response to nutrient	14/890	8,92E-06	0,003340354	0,003185348	TNC/TRPV1/TRIM24/HMOX1/CASTOR1/CIB2/GDAP1/CASTOR3/P2RY11/PDXP/FYN/PDK2/CASTOR2/VDR
GO:2001020	regulation of response to DNA damage stimulus	23/890	0,00023684	0,025621249	0,02443232	BCL2/MMS19/NSMCE1/ERCC1/NACC2/CHEK1/ABL1/NUDT16L1/PLA2R1/FAM168A/SLF1/ANKLE1/SPIRE2/BRCC3/FIGNL1/RFWD3/RAD51/RAD51AP1/FEM1B/DHX9/ATAD5/CHEK2/MAPT
GO:0034599	cellular response to oxidative stress	28/890	0,00062128	0,04800665	0,045778949	FOXO3/NCOA7/DAPK1/BCL2/TPM1/MAPKAP1/AKR1C3/PRKN/PRR5L/HMOX1/ABL1/PLA2R1/DHFR/SIRPA/FANCD2/MEAK7/MSRA/CDK2/ALDH3B1/CCNA2/NQO1/EZH2/FYN/EPAS1/PDK2/PPARGC1A/MAPT/SLC7A11
GO:0010212	response to ionizing radiation	17/890	0,00064734	0,049238598	0,046953729	BCL2/ERCC1/TRIM13/POLE3/FANCD2/TNKS1BP1/BRCC3/FIGNL1/RFWD3/RAD51/BLM/CRYAB/RAD51AP1/CHEK2/MSH2/BRCA2/XRCC2
**CBP TNF vs control TNF**						

GO:0031669	cellular response to nutrient levels	33/1185	1,76E-05	0,002427321	0,002084843	VDR/CADPS2/BCL2/HSPA5/WIPI1/GABARAPL1/ATF3/MDM2/TRPM4/CDKN1A/AKR1C3/CASTOR3/CIB2/GBA/CACNB3/PPM1D/P2RX4/FYN/TNC/NAMPT/IL15/RRAGD/TRPV1/CBL/GDAP1/EIF2AK2/NIM1K/SLC39A4/CASTOR1/RNF152/CD68/PDK2/PPARG
GO:0001101	response to acid chemical	41/1185	7,13E-05	0,007751608	0,006657912	PDGFRB/AKR1C1/WNT5B/RARB/DNMT1/CPEB3/TRIM16/PTGES/AKR1C3/DHFR/CASTOR3/LDLR/PPARGC1A/PDGFRA/NSMF/CEBPB/AKR1C2/NQO1/FYN/TNC/EDN1/CLDN1/CD38/SHMT1/CCN2/GCLM/ACER2/RRAGD/GLDC/TLR2/WNT5A/PRKCE/GDAP1/ID3/COL18A1/DHODH/CASTOR1/SPAAR/P2RY6/PPARG/TYMS
GO:0034599	cellular response to oxidative stress	37/1185	9,48E-05	0,009241083	0,007937232	FOXO3/PARP1/MAPKAP1/SRXN1/NCOA7/PRR5L/BCL2/DAPK1/MDM2/SLC7A11/TP53INP1/AKR1C3/DHFR/PPARGC1A/PLA2R1/SIRPA/IL6/PDGFRA/ALDH3B1/NQO1/FYN/ABCD1/ZNF580/ABL1/TPM1/GCH1/SOD3/EPAS1/TREX1/MSRA/RNF112/NR4A3/TXNRD1/PML/TLR6/PDK2/ATP2A2
GO:0062197	cellular response to chemical stress	39/1185	0,00045025	0,024702834	0,02121744	FOXO3/PARP1/MAPKAP1/SRXN1/NCOA7/PRR5L/BCL2/SLC25A23/DAPK1/MDM2/SLC7A11/TP53INP1/AKR1C3/DHFR/PPARGC1A/PLA2R1/SIRPA/IL6/PDGFRA/ALDH3B1/NQO1/FYN/ABCD1/ZNF580/ABL1/TPM1/GCH1/SOD3/EPAS1/TREX1/MSRA/RNF112/NR4A3/TXNRD1/PML/TLR6/PDK2/ATP2A2/PPARG
GO:0009411	response to UV	19/1185	0,00149805	0,048916582	0,042014802	PARP1/XPC/CHEK1/DTL/CREBBP/BCL2/MDM2/NOC2L/CDKN1A/TP53INP1/CDC25A/MSH2/POLH/RAD18/SDF4/TREX1/MME/PML/PRIMPOL
**p300 vs control**						
GO:0070482	response to oxygen levels	143/4777	1,32E-06	0,000704267	0,000608775	PSMD2/PDGFRB/PSMD3/PSMC4/LOXL2/PLOD1/ANGPTL4/TP53/EPAS1/ARNT2/CCN2/NR4A2/BCL2/EEF2K/PSMD1/AQP3/EP300/PSMC5/PSMC1/PSMD4/ATP6V1G1/BMP2/CPEB4/PSMD13/PSMA3/POLG/ATG7/PTGIS/ARNT/ACAA2/ITGA2/PSMB3/ELOC/USF1/ATF4/PAM/TM9SF4/PDK3/CAPN2/KCNK3/DDAH1/COL1A1/M

						ECP2/PSMA6/PMAIP1/PSMC2/PLAT/TGFBR3/LPAR1/CREB1/PRKCE/PTK2B/PSMA5/PPARG/PSMB6/BNIP3L/ATP6V0A2/ADM/NPPC/KCND2/ACTN4/SMAD3/MMP2/HP1BP3/ANG/PSMD11/ITPR2/FOXO3/CITED2/PSMB2/ENDOG/PSMB5/CHRNB2/POLB/EDN1/APAF1/PSMC6/THBS1/PLEKHN1/SCAP/NOTCH1/NKX3-1/VHL/TWIST1/TGFB1/DDIT4/PSMA4/CCDC115/PSMB8/TSC1/MTHFR/CXCL12/PSME1/KCNK2/PSMD14/VEGFA/ADA/HSD11B2/TGFBR2/TXN2/PSMA2/PSMD12/HDAC2/ERO1A/PLK3/EIF4EBP1/BMP7/FOXO1/ND5/CPEB1/DNMT3B/KCNMA1/ADAM8/VASN/LMNA/CRYAB/HK2/VCAM1/DPP4/CBL/AK4/CAV1/CCNA2/ROCK2/NDNF/ITPR1/PPARGC1A/NAMPT/ATP6V1A/PLAU/EGLN3/PSMB9/HIGD1A/SLC8A1/CYBA/SCFD1/NOX4/CYTB/EDNRA/ND4/UCP3/PSME2/PTGS2
GO:0062197	cellular response to chemical stress	129/4777	1,72E-06	0,000845569	0,000730918	DHRS2/HDAC6/OSER1/GSR/LRRC8E/ZNF622/PRKCD/BRF2/DAPK1/DNM2/TP53/EPAS1/PRDX1/NR4A2/BCL2/DNAJA1/SRXN1/LETM1/PPIF/SLC25A23/MGST1/MGMT/PRKN/GPX7/MAPK10/SOD1/LRRC8D/ATG7/ARNT/TXN/PRDX3/SPHK1/NFE2L1/ATF4/ADNP2/PRKD1/SERPINB6/CYP1B1/MYLK/CHD6/EGFR/MAPK9/ETV5/CCS/MSRB3/PPARG/NET1/MCL1/MMP2/HGF/CAPN3/MPV17/FANCC/RIPK1/FOXO3/PKD2/ENDOG/TPM1/TRPV4/PXN/ARL6IP5/NQO1/FYN/PTPRK/ZC3H12A/CDK2/NCOA7/FER/HSPA1B/KDM6B/CYCS/ARNTL/TSC1/EFHD1/AXL/AKR1C3/PRKRA/MSRB2/FBLN5/PCNA/MAPK13/MSRA/NME5/ALDH3B1/LRRC8C/NR4A3/NUDT2/MAPKAP1/HDAC2/EIF2S1/EZH2/ERO1A/PYROXD1/SIRPA/PLEKHA1/BMP7/FOXO1/TXNRD2/DNMT3B/GUCY1B1/STK26/GLRX2/TREX1/CD36/OXSR1/PDK2/IMPACT/VKORC1L1/RHOB/CAV1/MAPT/CCNA2/PNPT1/RELA/PPARGC1A/ERRFI1/FXN/KLF4/CYBA/MPO/GPX1/SLC12A6/NOX4/ABCD1/TNF/PTGS2/JAK2/SMPD3/PDGFD

GO:0042176	regulation of protein catabolic process	136/4777	6,43E-06	0,001844368	0,001594289	PSMD2/PSMD3/PSMC4/APC2/MYLIP/SH3D19/HSP90AB1/DAB2/FBXO22/PSME3IP1/PSMD1/ZFAND2A/PSMC1/PRKN/CAMLG/DDRGK1/YOD1/VCP/SNX9/ATG7/OPHN1/PDCL3/UQCC2/NFE2L1/GSK3A/LPCAT1/SMURF1/UBAC2/PSMC2/UBQLN4/EGFR/MAPK9/HSP90AA1/NUB1/CSNK1E/PHB/SUMO2/TIPARP/BCAP31/FURIN/PTK2B/OSBPL7/GJA1/TRIM32/TMTC3/CCAR2/TAF9/USP25/SMAD3/OS9/TIMP2/RNF217/RIC1/TMEM259/PRKACA/LDLR/USP5/SNRNP70/EZR/CLEC16A/MYCBP2/GRIN2C/RNFT1/OAZ2/FYN/NUPR1/SNX33/CSNK2A1/DTL/TRIB1/COMMD1/HSPA1B/ADRA2A/DACT1/SH3RF2/RNF19B/ARNTL/AXIN1/GPLD1/SGTA/PSME1/WWP2/CD81/PSMD14/HFE/SOCS5/MAD2L2/PTPN3/PTK2/DET1/AREL1/LRP1/SUFU/FAM83D/RNF180/ODC1/CCDC22/PLK3/BAG6/SERPINE2/MAD2L1/DISC1/BTRC/IRAK3/RCHY1/FOXO1/PSEN1/SENP1/ADAM8/EFNA1/ROCK1/ATP5IF1/FHIT/RILP/HGS/SOCS4/CAV1/TRIB3/HECW2/RELA/NRG1/PLEKHG5/FAF1/EEF1A2/TRIM67/PBK/USP14/C4BPB/GPX1/AGAP2/TNF/PSME2/SORL1/RNF144B/RHBDD3/GRIN2A
GO:0071479	cellular response to ionizing radiation	35/4777	1,46E-05	0,002611859	0,002257715	DNM2/SPIDR/SNAI2/TP53/TNKS1BP1/MGMT/YAP1/GRB2/RAD9A/CLOCK/POLE3/NET1/INTS7/MAPK14/TANK/TGFB1/TSPYL5/BCL2L1/KDM1A/GADD45A/SWI5/GTF2H5/NUCKS1/H2AX/TREX1/RAD51AP1/CRYAB/RHOB/MAP3K20/LIG4/NAMPT/FIGNL1/TMEM109/CYBA/NOX4
GO:0034599	cellular response to oxidative stress	110/4777	1,76E-05	0,002746439	0,002374048	DHRS2/HDAC6/OSER1/GSR/ZNF622/PRKCD/BRF2/DAPK1/DNM2/TP53/EPAS1/PRDX1/NR4A2/BCL2/SRXN1/PPIF/MGST1/MGMT/PRKN/GPX7/SOD1/ATG7/ARNT/TXN/PRDX3/SPHK1/NFE2L1/ATF4/ADNP2/PRKD1/CYP1B1/CHD6/EGFR/MAPK9/ETV5/CCS/MSRB3/NET1/MCL1/MMP2/HGF/MPV17/FANCC/RIPK1/FOXO3/PKD2/ENDOG/TPM1/PXN/ARL6IP5/NQO1/FYN/PTPRK/ZC3H12A/CDK2/NCOA7/FER/HSPA1B/KDM6B/CYCS/AR

						NTL/TSC1/AXL/AKR1C3/PRKRA/MSRB2/FBLN5/PCNA/MAPK13/MSRA/NME5/ALDH3B1/NR4A3/NUDT2/MAPKAP1/HDAC2/EIF2S1/EZH2/ERO1A/PYROXD1/SIRPA/PLEKHA1/BMP7/FOXO1/TXNRD2/GUCY1B1/STK26/GLRX2/TREX1/CD36/PDK2/IMPACT/VKORC1L1/RHOB/MAPT/CCNA2/PNPT1/RELA/PPARGC1A/FXN/KLF4/CYBA/MPO/GPX1/NOX4/ABCD1/TNF/JAK2/SMPD3/PDGFD
GO:0006970	response to osmotic stress	38/4777	5,29E-05	0,004927634	0,004259494	LRRC8E/HSP90AB1/TP53/LETM1/TSC22D3/SLC25A23/MAPK10/LRRC8D/ITGA2/SERPINB6/NOLC1/MYLK/EGFR/PAPPA2/PTK2B/LRRC8A/CAPN3/PKD2/FMO1/TSC22D4/TRPV4/HNMT/EFHD1/SORD/MAPK13/LRRC8C/MLC1/PLK3/ABCB1/KCNMA1/OXSR1/TLR3/ERRFI1/OXT/SLC12A6/CYTB/TNF/PTGS2
GO:0010498	proteasomal protein catabolic process	158/4777	0,00011880	0,007051071	0,006095013	PSMD2/PSMD3/PSMC4/KLHL15/RNF122/FBXL17/FBXL7/FBXW4/TBL1XR1/HSP90AB1/KCTD5/DAB2/FBXO22/PSME3IP1/TRIM2/HM13/UFD1/PSMD1/ZFAND2A/PSMC5/PSMC1/PSMD4/PSMD13/PRKN/FBXL2/CAMLG/DDRGK1/KCTD2/YOD1/VCP/PELI1/PSMA3/ANAPC16/OPHN1/FBXL4/GET4/NFE2L1/PSMB3/UBE2H/GSK3A/SMURF1/UBAC2/PSMA6/PMAIP1/PSMC2/UBQLN4/RHBDD1/FBXO45/MAPK9/NUB1/CSNK1E/CLOCK/SUMO2/BCAP31/FBXO17/PPP2R5C/DNAJC18/OSBPL7/NEMF/BUB3/PSMA5/PSMB6/TMTC3/FBXO38/CCAR2/FBXO33/TAF9/USP25/SDF2L1/OS9/RNF217/TMEM259/PRKACA/RMND5A/USP5/PTTG1/UBE2E1/PSMD11/PPP2CB/MTA1/CLEC16A/PSMB2/PSMB5/TOPORS/RNFT1/UGGT1/ARRB1/PSMC6/NUPR1/PRPF19/TRIB1/UBE2A/CDK2/FBXO6/COMMD1/HSPA1B/FOXRED2/SH3RF2/RNF19B/PSMA4/PSMB8/ARNTL/UBR3/SGTA/PSME1/WWP2/FBXL6/ANAPC2/PSMD14/CDC23/HFE/SOCS5/CDC34/PSMA2/UBXN11/DET1/ECPAS/AREL1/PSMD12/CUL5/RNF7/RNF180/HERC2/UBE2B/PLK3/BAG6/MAD2L1/BTRC/RCHY1/PSEN1/UBE2C/SENP1/DNAJC10/FHIT/UBXN6/UBE2D1/UGGT2/SEL1L/SOCS4/CAV1/

						TRIB3/HECW2/SPSB1/TMEM67/SYVN1/FAF1/PSMB9/PBK/KCTD13/USP14/FBXL19/KIF14/GPX1/RNF126/PSME2/TBL1X/RNF144B/NR1D1
GO:0000302	response to reactive oxygen species	85/4777	0,00011882	0,007051071	0,006095013	HDAC6/OSER1/PDGFRB/PRKCD/DNM2/PRDX1/BCL2/PPIF/SOD1/TXN/PRDX3/SPHK1/CAPN2/CYP1B1/COL1A1/EGFR/MAPK9/CCS/PTK2B/NET1/MMP2/HGF/MPV17/PPP2CB/FANCC/RIPK1/FOXO3/PKD2/STAT1/ENDOG/TPM1/PXN/EDN1/NQO1/AREG/FYN/PTPRK/BAK1/CDK2/FER/KDM6B/AXL/SESN3/HYAL2/AKR1C3/ADA/FBLN5/PCNA/MAPK13/NR4A3/HDAC2/EZH2/FOSL1/PLK3/SIRPA/PLEKHA1/BMP7/FOXO1/ND5/TXNRD2/ND6/GUCY1B1/STK26/GLRX2/TREX1/CD36/PDK2/CRYAB/IMPACT/RHOB/MAPT/CCNA2/RELA/PPARGC1A/SESN1/FXN/KLF4/SLC8A1/MPO/GPX1/FKBP1B/TNF/UCP3/SMPD3/PDGFD
GO:0001666	response to hypoxia	123/4777	0,00013742	0,007893034	0,006822813	PSMD2/PSMD3/PSMC4/LOXL2/PLOD1/ANGPTL4/TP53/EPAS1/ARNT2/NR4A2/BCL2/PSMD1/AQP3/EP300/PSMC5/PSMC1/PSMD4/BMP2/PSMD13/PSMA3/PTGIS/ARNT/ACAA2/ITGA2/PSMB3/ELOC/USF1/PAM/TM9SF4/PDK3/CAPN2/KCNK3/DDAH1/MECP2/PSMA6/PMAIP1/PSMC2/PLAT/TGFBR3/CREB1/PRKCE/PTK2B/PSMA5/PSMB6/BNIP3L/ADM/NPPC/KCND2/ACTN4/SMAD3/MMP2/HP1BP3/ANG/PSMD11/ITPR2/FOXO3/CITED2/PSMB2/ENDOG/PSMB5/CHRNB2/EDN1/APAF1/PSMC6/THBS1/PLEKHN1/SCAP/NOTCH1/NKX3-1/VHL/TWIST1/TGFB1/DDIT4/PSMA4/PSMB8/MTHFR/CXCL12/PSME1/KCNK2/PSMD14/VEGFA/ADA/HSD11B2/TGFBR2/TXN2/PSMA2/PSMD12/ERO1A/PLK3/EIF4EBP1/BMP7/ND5/CPEB1/DNMT3B/KCNMA1/ADAM8/VASN/LMNA/CRYAB/HK2/VCAM1/DPP4/AK4/CAV1/CCNA2/ROCK2/NDNF/ITPR1/PPARGC1A/PLAU/EGLN3/PSMB9/HIGD1A/SLC8A1/CYBA/SCFD1/NOX4/CYTB/EDNRA/ND4/UCP3/PSME2/PTGS2

GO:0006914	autophagy	163/4777	0,00014548	0,007895094	0,006824593	ATP6V0B/HDAC6/TBC1D5/TPCN1/HSPB8/DAPK1/EXOC4/VPS18/CDK5R1/TP53/PIM2/BCL2/PGAM5/C9orf72/ITGB4/TBC1D25/TRIM21/EP300/ATP6V1G1/ATG13/ATP6V1B2/PRKN/TRIM22/FBXL2/BCL2L11/SQSTM1/EI24/UVRAG/DRAM1/YOD1/ATG10/VCP/SBF2/ATG7/PTPN22/TOMM40/PLEKHF1/TOMM5/CALCOCO2/PRKD1/GSK3A/MAP1LC3B/SMURF1/MCOLN1/VPS13A/PIKFYVE/CHMP2A/UBQLN4/RMC1/HSP90AA1/ATG2A/HMGB1/ATG16L1/OSBPL7/IRGM/PACS2/VPS39/RRAGC/BNIP3L/ATP6V0A2/MCL1/RAB23/IFT88/SH3BP4/MARK2/HGF/KAT8/PRKACA/STX12/ULK3/SRPX/EPM2A/SNRNP70/SUPT5H/CLEC16A/VPS13D/VPS4A/FOXO3/HERC1/CHMP4C/PIP4K2C/CISD2/STBD1/PIP4K2A/VPS26A/RUBCNL/CDC37/LZTS1/NUPR1/CSNK2A1/LRSAM1/ZC3H12A/VPS37B/DHRSX/CHMP3/USP36/VPS41/TSC1/ATG4C/PRKAB1/SPATA18/SNAPIN/RRAGD/HSPA8/TMEM59/SESN3/ATP6V1D/AMBRA1/RAB5A/LAMTOR5/CHMP4A/NBR1/ATP6V0C/SREBF2/PTK2/RAB24/MAP1S/VTI1A/SYNPO2/DEPP1/ATG4D/PLK3/FZD5/RGS19/BMF/DAPK2/TBC1D17/FOXO1/PSEN1/ROCK1/ATP5IF1/UBXN6/HGS/HK2/LEPR/WASHC1/MAPT/TRIB3/ITPR1/PPARGC1A/SESN1/NAMPT/ATP6V1A/TICAM1/PRKAG3/MTERF3/VPS51/WIPI1/VPS13C/EEF1A2/TMEM150A/VPS37D/ARSB/ATP6V1C1/MTMR8/HDAC10/SCFD1/ACER2/RETREG1/MTCL1/EMC6/WDR24/HAP1
**p300 TNF vs control TNF**						
GO:0070482	response to oxygen levels	124/3597	3,63E-09	1,10E-06	8,95E-07	PDGFRB/PTGIS/PSMD2/EDN1/THBS1/PSMC4/BMP2/CCN2/ANGPTL4/CITED2/PSMD11/HMOX1/ARNT2/ANKRD1/PSMD3/CDKN1A/TP53/CPEB4/PPARG/BCL2/LPAR1/PSMD13/POLB/TGFB1/PSMC3/LOXL2/UBQLN1/PSMD1/VEGFA/PLOD1/PSMA7/DDAH1/AK4/PSMD4/ARNT/PSMC1/PSMB5/PSMD14/PSMB3/PSMB4/VASN/PSMC6/TWIST1/GATA6/PSMD6/HP1BP3/PSMC5/PSMC2/SIRT2/FOXO3/ITPR2/PSMB7/SLC11

						A2/CREB1/PSMA5/COL1A1/UBC/ANG/VCAM1/PSMB6/PSMD8/CXCL12/POLG/KCNK2/TM9SF4/NPEPPS/PSMB2/SMAD3/PLAT/ATP6V1G1/PDK3/KCND2/AQP3/ENDOG/EP300/ATG7/PSMB9/PLEKHN1/PSMA3/TEK/MDM2/CD34/PSMA6/CAV1/MMP2/CPEB1/EEF2K/REST/CCNA2/ACTN4/SLC2A1/PDK1/ITGA2/SCFD1/DPP4/PRKCE/PSME4/ACAA2/TLR2/PAM/PMAIP1/AGTRAP/DNMT3B/CCDC115/BRIP1/TGFBR2/ND4/MECP2/DDIT4/CBL/PSMD12/SLC8A1/SOD3/VEGFB/PSMA4/ND5/MYOCD/STC2/AJUBA/CD38/FAS/PGF/PCK1/HDAC2
GO:0001666	response to hypoxia	108/3597	4,61E-07	5,08E-05	4,14E-05	PTGIS/PSMD2/EDN1/THBS1/PSMC4/BMP2/ANGPTL4/CITED2/PSMD11/HMOX1/ARNT2/ANKRD1/PSMD3/TP53/BCL2/PSMD13/TGFB1/PSMC3/LOXL2/UBQLN1/PSMD1/VEGFA/PLOD1/PSMA7/DDAH1/AK4/PSMD4/ARNT/PSMC1/PSMB5/PSMD14/PSMB3/PSMB4/VASN/PSMC6/TWIST1/GATA6/PSMD6/HP1BP3/PSMC5/PSMC2/SIRT2/FOXO3/ITPR2/PSMB7/SLC11A2/CREB1/PSMA5/UBC/ANG/VCAM1/PSMB6/PSMD8/CXCL12/KCNK2/TM9SF4/NPEPPS/PSMB2/SMAD3/PLAT/PDK3/KCND2/AQP3/ENDOG/EP300/PSMB9/PLEKHN1/PSMA3/TEK/MDM2/CD34/PSMA6/CAV1/MMP2/CPEB1/REST/CCNA2/ACTN4/SLC2A1/PDK1/ITGA2/SCFD1/DPP4/PRKCE/PSME4/ACAA2/TLR2/PAM/PMAIP1/AGTRAP/DNMT3B/BRIP1/TGFBR2/ND4/MECP2/DDIT4/PSMD12/SLC8A1/SOD3/VEGFB/PSMA4/ND5/MYOCD/STC2/AJUBA/CD38/PGF/PCK1
GO:0062197	cellular response to chemical stress	103/3597	2,56E-06	0,000195588	0,000159471	BRF2/GSR/HMOX1/DAPK1/PRDX1/TP53/PRKD1/GUCY1B1/PPARG/HDAC6/BCL2/NQO1/SRXN1/UBQLN1/ZNF622/DHRS2/FBLN5/GPX8/ARNT/ABCD1/PXN/PKD2/SOD1/CAMKK2/LRRC8E/OSER1/RCSD1/LETM1/DNM2/MYLK/SIRPA/MAPK9/MAPK13/GPX1/SIRT2/FOXO3/PRKCD/EGFR/ATP13A2/HSPA1B/TMEM161A/SLC7A11/HSPA1A/PDGFD/MSRB3/MGMT/CHD6/NFE2L1/STK26/LRRC8D/FANCC/SERPINB6/MCL1/MAP1LC3A/CAPN3/DDIT3/FYN/MAPK10/ENDOG/ATG7/TXN/SLC25A23/VKORC1L1/KDM6B/RBM11/MDM2/CAV1/MMP2/PPIF/FER/REST/CCNA2/GPX7/AXL/SLC2A1/PDK1/DHFR/JAK2/TPM1/ALDH3B1/PRKN/SOD2/DNMT3B/MSRA/GPR37L1/MAP3K5/PYCR1/A

						RNTL/GCH1/SOD3/EIF2AK3/SLC12A6/PNPT1/TXNRD1/PYROXD1/MAPT/NCF2/NR4A3/NCOA7/NET1/FAS/ATP2A2/HDAC2
GO:0043618	regulation of transcription from RNA polymerase II promoter in response to stress	45/3597	3,05E-06	0,000224922	0,000183388	PSMD2/PSMC4/CITED2/PSMD11/HMOX1/PSMD3/TP53/PSMD13/PSMC3/PSMD1/ATF3/VEGFA/PSMA7/PSMD4/ARNT/PSMC1/PSMB5/PSMD14/PSMB3/PSMB4/PSMC6/PSMD6/PSMC5/PSMC2/SIRT2/PSMB7/PSMA5/UBC/PSMB6/PSMD8/HSPA1A/HSPA5/CHD6/PSMB2/ATF6/DDIT3/EP300/PSMB9/PSMA3/PSMA6/PSME4/HSF2/CREB3L1/PSMD12/PSMA4
GO:0010498	proteasomal protein catabolic process	127/3597	4,45E-05	0,001847669	0,001506482	TBL1XR1/PSMD2/PSMC4/PSMD11/KCTD5/PSMD3/VCP/UFD1/HM13/FBXL19/FBXL17/PSMD13/YOD1/PSMC3/UBQLN1/DAB2/TRIM3/UBE2H/PSMD1/PSMA7/DDRGK1/HSP90AB1/PSME3IP1/BCAP31/PSMD4/RNF7/PSMC1/PSMB5/PSMD14/TRIM2/PSMB3/PSMB4/HERPUD1/FBXW4/CLOCK/TMTC3/PSMC6/UGGT1/ANAPC16/KLHL15/PSMD6/RNFT1/HECW2/MAPK9/PSMC5/GPX1/PSMC2/SIRT2/NSFL1C/PELI1/ZFAND2A/CSNK1E/HSPA1B/PSMB7/CDC34/FBXO38/PSMA5/FBXL4/CUL1/PPP2R5C/UBC/USP5/KCTD2/UBAC2/PSMB6/PSMD8/HSPA1A/ANAPC2/OSBPL7/UBE2D1/HSPA5/RHBDD1/CAMLG/FBXL7/DET1/GCLC/SPSB3/NFE2L1/FBXO22/SOCS5/PSMB2/NUB1/DDIT3/TRIB1/RNF14/GSK3A/CEBPA/UGGT2/PSMB9/PSMA3/MDM2/PSMA6/CAV1/PKD1/RNF122/MTA1/ERLIN1/FBXL2/SH3RF2/UBXN11/GET4/RNF121/PSME4/FBXO33/DERL3/PMAIP1/PRKN/COMMD1/PRKACA/SENP1/PLAA/RMND5A/USP13/IL33/NUPR1/RNF180/PSMD12/ARNTL/FBXO17/PSMA4/SPSB4/LRRC29/TBX21/PBK/HECW1/MAP1A/PLK1
GO:0034599	cellular response to oxidative stress	86/3597	6,21E-05	0,002387555	0,001946673	BRF2/GSR/HMOX1/DAPK1/PRDX1/TP53/PRKD1/GUCY1B1/HDAC6/BCL2/NQO1/SRXN1/UBQLN1/ZNF622/DHRS2/FBLN5/GPX8/ARNT/ABCD1/PXN/PKD2/SOD1/CAMKK2/OSER1/DNM2/SIRPA/MAPK9/MAPK13/GPX1/SIRT2/FOXO3/PRKCD/EGFR/ATP13A2/HSPA1B/TMEM161A/SLC7A11/HSPA1A/PDGFD/MSRB3/MGMT/CHD6/NFE2L1/STK26/FANCC/MCL1/MAP1LC3A/FYN/ENDOG/ATG7/TXN/VKORC1L1/KDM6B/RBM11/MDM2/MMP2/PPIF/FER/REST/CCNA2/GPX7/AXL/PDK1/DHFR/JAK2/TPM1/ALDH3B1/PRKN/SOD2/MS

						RA/GPR37L1/MAP3K5/PYCR1/ARNTL/GCH1/SOD3/PNPT1/TXNRD1/PYROXD1/MAPT/NCF2/NR4A3/NCOA7/NET1/ATP2A2/HDAC2
GO:0043161	proteasome-mediated ubiquitin-dependent protein catabolic process	110/3597	0,00025109	0,006669281	0,005437743	TBL1XR1/PSMD2/PSMC4/PSMD11/KCTD5/PSMD3/VCP/UFD1/FBXL19/FBXL17/PSMD13/YOD1/PSMC3/UBQLN1/DAB2/TRIM3/UBE2H/PSMD1/PSMA7/DDRGK1/HSP90AB1/BCAP31/PSMD4/RNF7/PSMC1/PSMB5/PSMD14/TRIM2/PSMB3/PSMB4/HERPUD1/FBXW4/CLOCK/PSMC6/ANAPC16/KLHL15/PSMD6/HECW2/MAPK9/PSMC5/PSMC2/SIRT2/NSFL1C/PELI1/ZFAND2A/CSNK1E/HSPA1B/PSMB7/CDC34/FBXO38/PSMA5/FBXL4/CUL1/PPP2R5C/UBC/USP5/KCTD2/PSMB6/PSMD8/HSPA1A/ANAPC2/UBE2D1/HSPA5/CAMLG/FBXL7/DET1/GCLC/SPSB3/FBXO22/SOCS5/PSMB2/NUB1/DDIT3/TRIB1/RNF14/GSK3A/CEBPA/PSMB9/PSMA3/MDM2/PSMA6/CAV1/RNF122/MTA1/ERLIN1/FBXL2/SH3RF2/UBXN11/RNF121/PSME4/FBXO33/DERL3/PRKN/COMMD1/SENP1/PLAA/RMND5A/IL33/RNF180/PSMD12/ARNTL/FBXO17/PSMA4/SPSB4/LRRC29/TBX21/PBK/HECW1/MAP1A/PLK1
GO:0032436	positive regulation of proteasomal ubiquitin-dependent protein catabolic process	29/3597	0,00035080	0,008467324	0,006903763	VCP/UBQLN1/DAB2/DDRGK1/BCAP31/HERPUD1/MAPK9/SIRT2/ZFAND2A/CSNK1E/HSPA1B/USP5/HSPA1A/DET1/GCLC/FBXO22/SOCS5/NUB1/TRIB1/RNF14/GSK3A/CEBPA/MDM2/CAV1/SH3RF2/PRKN/IL33/RNF180/PLK1
GO:0042176	regulation of protein catabolic process	96/3597	0,00242499	0,032003846	0,026094072	PSMD2/PSMC4/PSMD3/VCP/YOD1/UBQLN1/DAB2/PSMD1/LPCAT1/TIPARP/DDRGK1/HSP90AB1/PSME3IP1/BCAP31/PSMC1/DACT1/PSMD14/HERPUD1/SERPINE2/TMTC3/RNFT1/HECW2/MAPK9/GPX1/PSMC2/SIRT2/ZFAND2A/EGFR/ATP13A2/CSNK1E/HSPA1B/PHB/FURIN/HGS/TIMP2/RIC1/PCSK9/ADRA2A/USP5/UBAC2/HSPA1A/OSBPL7/PLEKHG5/CCDC22/HSP90AA1/CAMLG/DET1/TRIM32/GCLC/MYLIP/SH3D19/NFE2L1/FBXO22/SOCS5/LAPTM4B/SMAD3/ODC1/NUB1/FYN/TRIB1/RNF14/GSK3A/CEBPA/ATG7/MDM2/CAV1/PKD1/EZR/PTK2/SH3RF2/IRAK3/DERL3/WNT5A/PRKN/COMMD1/PRKACA/SENP1/USP

						13/WDR91/IL33/NUPR1/RNF180/PTPN3/ARNTL/GJA1/AMER1/APC2/PBK/SUFU/GRIN2C/IL1B/SOX17/HECW1/MAP1A/PLK1/CDH1
GO:1905898	positive regulation of response to endoplasmic reticulum stress	15/3597	0,00310562	0,038757486	0,031600597	UBQLN1/PTPN1/BCAP31/TMEM33/HERPUD1/RNFT1/PPP1R15A/ATF6/DDIT3/CAV1/PIK3R1/PMAIP1/BAK1/USP13/PTPN2
